# Identification of Immunoreactive *Leishmania infantum* Protein Antigens to Asymptomatic Dog Sera through Combined Immunoproteomics and Bioinformatics Analysis

**DOI:** 10.1371/journal.pone.0149894

**Published:** 2016-02-23

**Authors:** Maria Agallou, Evita Athanasiou, Martina Samiotaki, George Panayotou, Evdokia Karagouni

**Affiliations:** 1 Laboratory of Cellular Immunology, Department of Microbiology, Hellenic Pasteur Institute, Athens, Greece; 2 Chemical Engineering Laboratory, Department of Analysis, Design and Control of Chemical Processes, Department of Chemical Engineering, Aristotle University of Thessaloniki, Thessaloniki, Greece; 3 Biomedical Sciences Research Center “Alexander Fleming,” Vari, Greece; Department of Medical Lab Technology, Faculty of Applied Medical Sciences, Taibah University, SAUDI ARABIA

## Abstract

*Leishmania infantum* is the etiologic agent of zoonotic visceral leishmaniasis (VL) in countries in the Mediterranean basin, where dogs are the domestic reservoirs and represent important elements in the transmission of the disease. Since the major focal areas of human VL exhibit a high prevalence of seropositive dogs, the control of canine VL could reduce the infection rate in humans. Efforts toward this have focused on the improvement of diagnostic tools, as well as on vaccine development. The identification of parasite antigens including suitable major histocompatibility complex (MHC) class I- and/or II-restricted epitopes is very important since disease protection is characterized by strong and long-lasting CD8^+^ T and CD4^+^ Th1 cell-dominated immunity. In the present study, total protein extract from late-log phase *L*. *infantum* promastigotes was analyzed by two-dimensional western blots and probed with sera from asymptomatic and symptomatic dogs. A total of 42 protein spots were found to differentially react with IgG from asymptomatic dogs, while 17 of these identified by Coommasie stain were extracted and analyzed. Of these, 21 proteins were identified by mass spectrometry; they were mainly involved in metabolism and stress responses. An *in silico* analysis predicted that the chaperonin HSP60, dihydrolipoamide dehydrogenase, enolase, cyclophilin 2, cyclophilin 40, and one hypothetical protein contain promiscuous MHCI and/or MHCII epitopes. Our results suggest that the combination of immunoproteomics and bioinformatics analyses is a promising method for the identification of novel candidate antigens for vaccine development or with potential use in the development of sensitive diagnostic tests.

## Introduction

*Leishmania* is an obligate intracellular protozoan parasite that is transmitted via phlebotomine sand flies. It causes a broad spectrum of human diseases ranging from self-healing cutaneous lesions to severe visceral dissemination. Visceral leishmaniasis (VL) is the most severe clinical form, giving rise to approximately 500,000 new cases each year, and is usually fatal if not treated properly [1]. Drugs used to treat zoonotic VL (ZVL) are not sufficiently effective to eradicate disease owing to the development of resistant parasitic strains and still the epidemiological risk persists [2,3]. In southern Europe and the Mediterranean region, ZVL is the most widespread form of leishmaniasis caused by a single parasite species, *Leishmania (L*.*) infantum*, with dogs representing the main domestic reservoirs of the parasite and playing significant role in disease transmission. Among Mediterranean countries, Greece is considered highly endemic with high rates of canine seropositivity in various geographical areas and the major focal areas of human VL exhibit a high prevalence of seropositive dogs [4,5,6,7]. Accordingly, the control of ZVL considerably reduces the number of reservoirs, and consequently, the infection rate in humans [8].

In this context, immunoprophylaxis has promoted the development of improved vaccine as an important tool to control the disease and subsequently decrease the infection pressure of *L*. *infantum* in humans [9]. However, *Leishmania* vaccinology still suffers from several bottlenecks that limit progress toward the development of effective and universal vaccines. Among them are the high levels of genetic diversity in parasites and the difficulty in identifying surrogate markers of resistance in naturally immunized hosts. Until now, the existing vaccines developed against ZVL gave controversial results in terms of protective efficacy. Specifically, two vaccines have been developed in Brazil (Leishmune^®^ and Leish-Tec^®^) and only one in Europe (CaniLeish^®^). Leish-Tec^®^ consisted by recombinant amastigote A2 antigen led to significant protective efficacy (71%) [10]. Leishmune^®^ and CaniLeish^®^ composed of fucose-mannose ligand (FML) antigen of *L*. *donovani* promastigotes or *L*. *infantum* secreted/excreted promastigote antigens, respectively, showed to confer significant protection against disease [11,12]. Nowadays, in Brazil the vaccine Leishmune^®^ has been withdrawn, since there are reservations regarding the efficacy and only Leish-Tec^®^ is commercially available [13].

Currently, in order to define antigens able to elicit protective immune responses for potential use as vaccine candidates, it is advisable to search for immunoreactive proteins to seropositive but disease-free hosts. Asymptomatic dogs are broadly divided into two categories: the ones that are susceptible to disease and factors such as immunosuppresion or concomitant diseases could lead them to the progression of the clinical disease and death if not cured, and the ones resistant to the parasite [14]. The second group is generally characterized as exposed/infected dogs without or with low levels of antileishmanial antibodies and negative or positive PCR [12,14]. According to a recent study, knowledge of immunodominant serospecificities in such asymptomatic dogs could lead to the identification of *Leishmania* antigens that could be used as potential vaccine candidates, since IgG-dominated antibody responses depend on T cell help. Further, these antigens could be instrumental in immune monitoring in the discrimination of asymptomatic dogs through the development of new diagnostic tools [15].

Taking the above into consideration, in the present study, a comparative immunoproteomics analysis of *L*. *infantum* protein extracts using sera from asymptomatic and symptomatic dogs obtained from an endemic area in Greece was conducted to identify molecules that are specifically recognized by antibodies found exclusively in asymptomatic hosts. Further, identified immunogenic molecules were subjected to an *in silico* analysis to investigate the existence of potential MHC class I- and MHC class II-restricted epitopes that are crucial for the efficient activation of CD8^+^ and CD4^+^ T cell populations. This combined approach enabled the identification of several immunogenic proteins that could be used for the development of an effective vaccine against VL.

## Materials and Methods

### Ethics statement

The collection of biological samples from domestic dogs (*Canis familiaris*) was conducted by licensed veterinarians previously informed about the purposes of the study during a workshop carried out at Hellenic Pasteur Institute. Volunteer veterinarians have been assigned to examine the dogs and to collect the biological samples (blood and bilateral eyelid specimen) in addition to standard veterinary care after obtaining written consent from dog owners for participation in the study. Owners were previously informed of the purpose of the study, the confidentiality of the data, and their voluntary participation. All animals were humanely treated during sample collection. Dogs were manually restrained during blood draws and bilateral eyelid specimen collection, in accordance with National Law 160/91 (FEK 64/A/91), which adheres to the European Directive 86/609/EEC on the approximation of laws, regulations, and administrative provisions of the Member States regarding the protection of animals used for experimental and other scientific purposes. According to this legislation, no special permission is required for the use of animals subjected to clinical examination and biological sample collection for diagnostic purposes. In addition, this study was also approved by the Hellenic Pasteur Institute Animal Bioethics Committee regulations according to the EU Directive 2010/63 and the National Law 2013/56.

### Serum samples

Serum samples (n = 112) from domestic adult dogs (*C*. *familiaris*) that varied in age, weight, and breed were used. Samples were collected between June and September 2012 from the Attiki area in Greece, which is an endemic region for canine VL (CVL). The dogs enrolled in the study were negative for ehrlichiosis and dirofilariasis. Additionally, none of the dogs were previously vaccinated with the commercially available vaccine in Europe, CaniLeish^®^. Dogs were examined by observing the typical clinical signs of CVL (ocular and/or skin lesions, onychogryphosis, progressive weight loss, muscular atrophy, epistaxis, apathy, and generalized lymphadenomegaly, among others) and were subjected to serological (immunofluorescence antibody tests [IFAT] and enzyme-linked immunosorbent assays [ELISA]) and molecular (real-time PCR) diagnostic tests to verify infection by *L*. *infantum*. Hematological and biochemical analyses for other clinicopathological findings were also performed. IFAT was carried out using *L*. *infantum* promastigote antigens, as described previously [16]. Antibody titers greater than or equal to 1:200 were considered positive for CVL, while an antibody titer of 1:100 was considered borderline and owners were kindly asked to have their dogs re-tested after one month. ELISA was performed to detect specific antibodies against *L*. *infantum*, according to a previously described method [6]. The cut-off value of ELISA was determined with the use of serum samples from healthy dogs (n = 11) living in a non-endemic area (Bristol, UK), kindly provided by Dr. Tasker (School of Veterinary Sciences, University of Bristol). TaqMan chemistry was used to detect *L*. *infantum* DNA in peripheral blood or bilateral eyelid specimens, as described by Mary et al. [17], with some modifications. Briefly, PCR primers and a hydrolysis probe (TaqMan MGB probe) targeting a 145-bp region of the *L*. *infantum* kinetoplast minicircle DNA were synthesized by Eurofins Genomics-VBC Biotech (Vienna, Austria). The sequences of the primers and the TaqMan probe were 5′-CTTTTCTGGTCCTCCGGGTAGG-3′ (RV1, forward primer), 5′-CCACCCGGCCCTATTTTACACCAA-3′ (RV2, reverse primer), and FAM-TTTTCGCAGAACGCCCCTACCCGC-BHQ (TaqMan probe). Each amplification was performed in triplicate in a 20-μl reaction mixture containing 1× KAPA Probe Fast qPCR Master Mix (Kapa Biosystems, Wilmington, MA, USA), 15 pmol forward primer, 5 pmol reverse primer and 25 pmol TaqMan probe. The thermal cycling profile was 95°C for 5 min, followed by 45 cycles at 95°C for 30 s and 57.6°C for 45 s. Finally, animals were classified as healthy, asymptomatic, or symptomatic according to Solano et al. [14,18]. Healthy dogs (n = 52) did not present clinical signs and/or clinicopathological abnormalities and were characterized as *Leishmania*-negative based on serological and molecular diagnostic tests. Symptomatic dogs were characterized as animals with IFAT titers of greater than 1:200, positive ELISA and PCR results, and more than 2 clinical symptoms (n = 13). Clinically healthy animals with low antibody titers, positive or negative based on PCR, were considered asymptomatic (n = 47). These dogs were subjected to complete clinical examinations and serological tests periodically for 2 years, a timeframe within they could develop disease, to verify that they remained asymptomatic. From this group, 9 serum samples were selected, since they did not show any progression of infection in terms of antibody titers elevation and presence of any kind of disease symptoms during the 2-years period. Also, from the group of symptomatic dogs, 5 serum samples were selected based on high IFAT titer (≥1600) and presence of symptoms, correlated with established infection (S1 Table). Consequently, for 2D western blots, serum samples obtained from each group (asymptomatic: n = 9; symptomatic: n = 5) were pooled in equal ratios by volume.

### Parasites

*L*. *infantum* (MCAN/PT/98/IMT 244; Zymodeme MON-1) promastigotes were used until the 5^th^ *in vitro* culture passage to maintain parasite infectivity. Parasites were cultured in RPMI-1640 medium (Biochrom AG, Berlin, Germany) supplemented with 2 mM l-glutamine, 10 mM HEPES, 24 mM NaHCO_3_, 100 U/ml penicillin, 100 μg/ml streptomycin, and 10% heat-inactivated fetal bovine serum (FBS; Biochrom AG) at 26°C. For the experiments, parasites were harvested at the late-log growth phase, since this parasite form is commonly used in serodiagnosis of the disease and it contains increased amount of infective forms [19].

### Sample preparation

Sample preparation was performed as described by Bente et al. [20]. Briefly, parasites (3 × 10^8^) were washed three times with Hank’s Balanced Salt Solution supplemented with 0.1% w/v glucose and subjected to centrifugation at 500 × *g* for 15 min at 20°C. The pellets were resuspended in lysis buffer solution (40 mM Tris [pH 9.5], 1 mM EDTA, 0.01 mM E-64, and 3 mM phenanthroline), subjected to five freeze–thaw cycles (N_2_/37°C), and centrifuged at 17,000 × *g* for 20 min at 4°C. The supernatant was collected and the protein content was determined using the MicroBCA Assay (Pierce, Rockford, IL, USA).

### One-dimensional gel electrophoresis (1-DE) and immunoblotting

The whole-cell protein preparation (200 μg) was separated by 1-dimensional 12% sodium dodecyl sulfate-polyacrylamide gel electrophoresis (SDS-PAGE; 1-DE), as described by Laemmli [21], using the Mini-Protein II System (Bio-Rad, Hercules, CA, USA) at 200 V. Proteins were electroblotted onto nitrocellulose membranes. Blotted membranes were blocked with 5% non-fat dried milk in TBS-T for 1 h at room temperature and were then incubated overnight at 4°C with serum samples from each dog that were diluted 1:500 or 1:1,000. Membranes were washed 3 times with TBS-T and incubated with rabbit peroxidase-conjugated anti-dog IgG (Cat. No. A9042, Sigma, St. Louis, MO, USA), IgG1 (Cat. No. AHP947P, AbD Serotec, Kidlington, UK) or IgG2 (Cat. No. AHP948P, AbD Serotec) at a dilution of 1:10,000 for 1 h. Reactive bands were detected using Metal Enhanced DAB reagent (Thermo Scientific, Rockford, IL, USA). After optimization experiments based on reactivity and specificity, asymptomatic dog serum samples were used at a final dilution of 1:500 and symptomatic dog serum samples were used at a final dilution of 1:1,000 (S1 Fig).

### Two-dimensional gel electrophoresis (2-DE)

Rehydration buffer (200 μl) was added to promastigote solubilized extracts [8 M urea, 2 M thiourea, 4% (w/v) CHAPS, 2.4% (w/v) aminosulphobetaine-14 (w/v) (ASB-14), 1% (w/v) dithiothreitol (DTT), and 0.2% (v/v) Biolytes (Bio-Rad)] and samples were incubated for 30 min at room temperature, followed by centrifugation (17,000 × *g*, 40 min). For electrophoresis in the first-dimension, 150 μl of cell extract was applied to IPG strips (7 cm, pH 3–10 or pH 5–8; Bio-Rad) and allowed to rehydrate for 18–22 h under a maximum current of 50 μA per strip using the PROTEAN IEF Cell (Bio-Rad). Isoelectric focusing was performed at 250 V for 15 min and at 4,000 V for 20,000 Vh. Subsequently, the IPG strips were reduced (130 mM DTT) and then alkylated (135 mM iodoacetamide) for 15 min in equilibration buffer [6 M urea, 0.375 M Tris-HCl (pH 8.8), 2% (w/v) SDS, 20% (v/v) glycerol]. IPG strips were transferred to a 12% SDS-PAGE gel and sealed with 1% (w/v) agarose for electrophoresis in the second dimension. Protein separation was performed using the Mini-Protein II System (Bio-Rad) at 200 V, until the dye front reached the bottom of the gel. After electrophoresis was complete, gels were stained with Coomassie Brilliant Blue G-250 (Bio-Rad) for 12 h at room temperature. Destaining was performed with subsequent washes using 25% methanol for 6 h. The gels were stored in 25% (w/v) ammonium-sulfate at 4°C. The reproducibility of a 2D pattern was confirmed when three consecutive runs produced identical patterns.

### Western blotting

To identify reactive spots recognized by the antibodies present in dog sera, the 2-DE proteins were transferred to nitrocellulose membranes (330 mA, 2 h) and were stained with Ponceau S. Membranes were blocked with 5% non-fat dried milk in TBS-T for 1 h at room temperature. Next, the membranes were washed 3 times (5 min each) with TBS-T and probed with pools of sera from symptomatic (n = 5; 1:1,000) or asymptomatic (n = 9; 1:500) dogs overnight at 4°C (S1 Table). Membranes were incubated with anti-dog IgG-HRP secondary antibody (1:10,000; Sigma) for 1 h at room temperature. After three washes with TBS-T, immunoblots were developed using Metal Enhanced DAB reagent for 1–3 min and the reaction was stopped by replacing the developing solution with water. The stained gels and blots were scanned and analyzed with PDQuest v8.0 (Bio-Rad), followed by an additional visual analysis by three independent investigators in order to select spots recognized exclusively by asymptomatic dog sera.

### Selection of protein spots

To select spots that were recognized only by asymptomatic dog sera, three independent protein separations, each obtained from independent parasite cultures, were performed. To select and identify these spots, the blots from asymptomatic and symptomatic dogs were aligned and only those spots that were detected in the blots of asymptomatic dogs were selected. To match the antigen spots in western blots with the corresponding protein spot in the Coomassie-stained gel, the blot coordinates were defined by aligning the Ponceau S staining pattern of the blot filter with the Coomassie-stained gel. Blot spots with perfect overlap were excised manually from the gels for protein identification.

### In-gel digestion of proteins with trypsin

Protein digestion was performed according to standard procedures [22]. Briefly, the gel pieces were destained, dehydrated with acetonitrile (ACN), and then rehydrated with 25 mM ammonium bicarbonate (NH_4_HCO_3_) buffer. Subsequently, gel pieces were treated with 25 mM NH_4_HCO_3_ buffer containing 10 mM DTT for 45 min at 56°C followed by incubation in the dark with 50 mM iodoacetamide in NH_4_HCO_3_ buffer. After dehydration with ACN, the gel pieces were rehydrated and dehydrated again, and finally rehydrated with 12.5 ng/μl trypsin (Trypsin Gold; Promega, Madison, WI, USA) in 25 mM NH_4_HCO_3_ buffer. After overnight incubation at 37°C, peptides were extracted from the gel pieces with ACN containing 5% formic acid and dried in a vacuum centrifuge. The samples were reconstituted in 2% (v/v) ACN/0.1% (v/v) formic acid and sonicated in a water bath for 5 min.

### LC-MS/MS analysis

The purified peptides were analyzed by HPLC MS/MS (high-performance liquid chromatography and tandem mass spectrometry) using a C-18 column coupled to an LTQ Orbitrap XL Mass Spectrometer (Thermo Scientific). Ten microliters of the peptide mixture was pre-concentrated at a flow-rate of 5 μl/min for 10 min using a C18 trap column and then loaded onto a 50-cm C18 column (75 μm ID, particle size 2 μm, 100 Å, Acclaim PepMap RSLC, Thermo Scientific). The binary pumps for HPLC (RSLCnano, Thermo Scientific) contained solution A (2% (v/v) ACN in 0.1% (v/v) formic acid) and solution B (80% ACN in 0.1% formic acid). The peptides were separated using a linear gradient of 4–40% B for 55 min at a flow rate of 300 nl/min. The column was placed in an oven at 35°C. Full-scan MS spectra were acquired in the orbitrap (m/z 300–1600) in profile mode and data-dependent acquisition with the resolution set to 60,000 at m/z 400 and an automatic gain control target of 10^6^. The six most intense ions were sequentially isolated for collision-induced MS/MS fragmentation (normalized CID of 35%) and detection in the linear ion trap. Dynamic exclusion was set to 60 sec. Ions with single charge states were excluded. A lock-mass of m/z 445,120025 was used for internal calibration. The software Xcalibur (Thermo Scientific) was used to control the system and to acquire raw files.

### Data analysis and database search

Protein identification was performed using Proteome Discoverer 1.4 (Thermo Scientific) and the SEQUEST HT search engine. The Orbitrap raw data were searched against the LinfantumAnnotatedProteins_TriTrypDB-4.2 FASTA database (http://tritrypdb.org). Search parameters included a molecular weight ranging from 350 to 5,000 Da, an S/N threshold of 1.5, a precursor mass tolerance of 10 ppm, an MS/MS fragment tolerance of 0.6 Da, a maximum of two missed cleavages by trypsin, and methionine oxidation and acetylation of the N-terminus as variable modifications. Search results were filtered with XCorr (+2 ≥ 2.0 and +3 ≥ 2.5). The criteria used to accept protein identification included the high-confidence single-threshold filter of the cross-correlation score versus charge state, the extent of sequence coverage, and the number of peptides that matched. Three different LC-MS/MS analyses were performed on the isolated spots and the proteins included were identified in two of the three LC-MS/MS runs.

### Prediction of MHC class I- and II-restricted epitopes

To map the promising epitopes from the identified *L*. *infantum* proteins, a combination of algorithms that predict peptide binding to human HLA were used since there are no algorithms that predict potential dog MHC class I and MHC class II binders and because major sequence similarity exists between human and dog HLA [23]. Specifically, protein sequences were screened individually for the best MHC class I- and MHC class II-binding epitopes using the NetCTL (http://www.cbs.dtu.dk/services/NetCTL/) and SYFPEITHI (http://www.syfpeithi.de) or NetMHCII (http://www.cbs.dtu.dk/services/NetMHCII/) and SYFPEITHI algorithms, respectively. NetCTL version 1.2 predicts peptides that are restricted to HLA class I supertypes, as well as predicted HLA binding, proteasomal C-terminal cleavage, and transport efficiency with antigen processing (TAP) molecules. The prediction for SYFPEITHI is based on published motifs from natural ligands and considers the amino acids in the anchor and auxiliary anchor positions, as well as other frequent amino acids [24]. NetMHCII version 2.2 predicts the binding of peptides to specific HLA class II alleles using artificial neuron networks. Protein sequences were screened against the most frequent HLA alleles in the human population, specifically HLA-A2, HLA-A24, and HLA-B7 for MHC class I and the HLA-DRB supertype for MHC class II. The cut-off score was adjusted to ≥20 for SYFPEITHI and a default prediction threshold (>0.75) indicating an accuracy of >85% was used for NetCTL. For NetMHCII, extracted peptides were classified into weak binders (binding affinity ≤ 500 nM) or strong binders (binding affinity ≤ 50 nM). For each method, peptides were tested and ranked according to their scores, and higher ranks indicated better binders. Based on the prediction results using the algorithms, 9-mer MHC class I-restricted epitopes and 15-mer MHC class II-restricted epitopes with the highest predicted affinities that were recognized by more than two HLA alleles were extracted (S2 and S3 Tables).

### Antigenicity evaluation

Protein antigenicity was calculated using the VaxiJen and ANTIGENpro servers, which predict whether a protein is a protective antigen using alignment-free approaches. VaxiJen v2.0 (http://www.ddg-pharmfac.net/vaxijen) is based on the auto cross-variance transformation of protein sequences into uniform vectors of principal amino acid properties. The accuracy of the server for parasites reaches 78% at a threshold of 0.5 [25]. ANTIGENpro (http://scratch.proteomics.ics.uci.edu/) uses protein antigenicity microarray data to predict antigenicity. The accuracy of this server is 76% according the results of cross-validation experiments [26]. A protein was considered a protective antigen when it scored greater than 0.5 using both servers.

## Results

### Differential profile of IgG serum patterns between asymptomatic and symptomatic dogs

**Fig 1:**
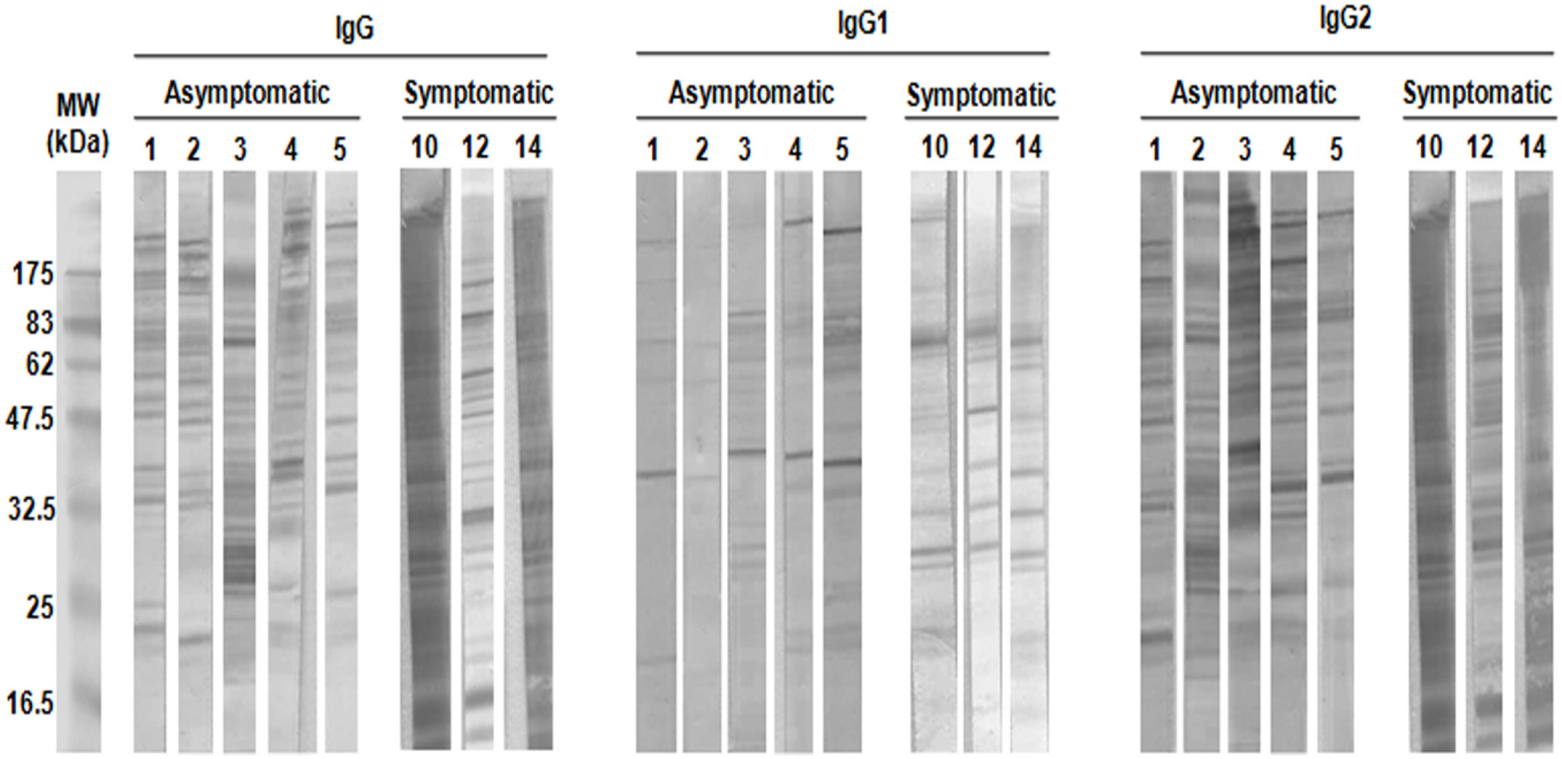
IgG reaction pattern associated with CVL status. Total *Leishmania infantum* promastigote cell extracts were separated with 12% SDS-PAGE and electrotransferred to nitrocellulose membranes. The membranes were probed with serum obtained from individual asymptomatic dogs (1:500) or symptomatic dogs (1:1,000) and secondary anti-dog (A) IgG, (B) IgG1 or (C) IgG2 (1:10,000). Representative western blots from each group of dogs are depicted (asymptomatic: n = 5; symptomatic: n = 3). Numbers represent the code numbers of the dogs enrolled in the study. Molecular weight markers are shown on the left-hand side.

Levels of anti-*Leishmania* specific IgG subclasses in dogs have been suggested as a marker for the susceptibility and consequent clinical status of the infected animals, with IgG1 subtype levels elevated in the presence of symptomatology and severe disease [11,27,28]. To gain insight into IgG subclasses pattern induced in the two groups included in the present study, parasite extracts in the respective western blots were probed with serum dilutions from individual asymptomatic dogs (n = 9; 1:500) and symptomatic dogs (n = 5; 1:1,000). The blots obtained revealed a broad range of immune specificities and extensive heterogeneity of the serological anti-parasite responses between individual dogs (Fig 1). Determination of IgG subclasses responses showed that IgG2 response contributed more than IgG1 to the immunoblots observed to total IgG in the symptomatic dogs, while in asymptomatic dogs IgG immunodetection patterns were attributed almost to IgG2 response with a very weak to scarce IgG1 reactivity restricted to a small region, with a main protein band of 40 kDa (Fig 1). This response profile was further confirmed by ELISA, with an elevated IgG2/IgG1 ratio detected in asymptomatic compared to symptomatic dogs (S1 Table). Given the hypothesis that IgG response is a direct effect of T-cell activation and a possible biomarker of resistance in asymptomatic infected dogs, sera from this group were used for the detection of immunodominant antigens reactive exclusively to asymptomatic dog sera.

### Characterization of 2D protein profiles of *L*. *infantum* promastigotes

**Fig 2:**
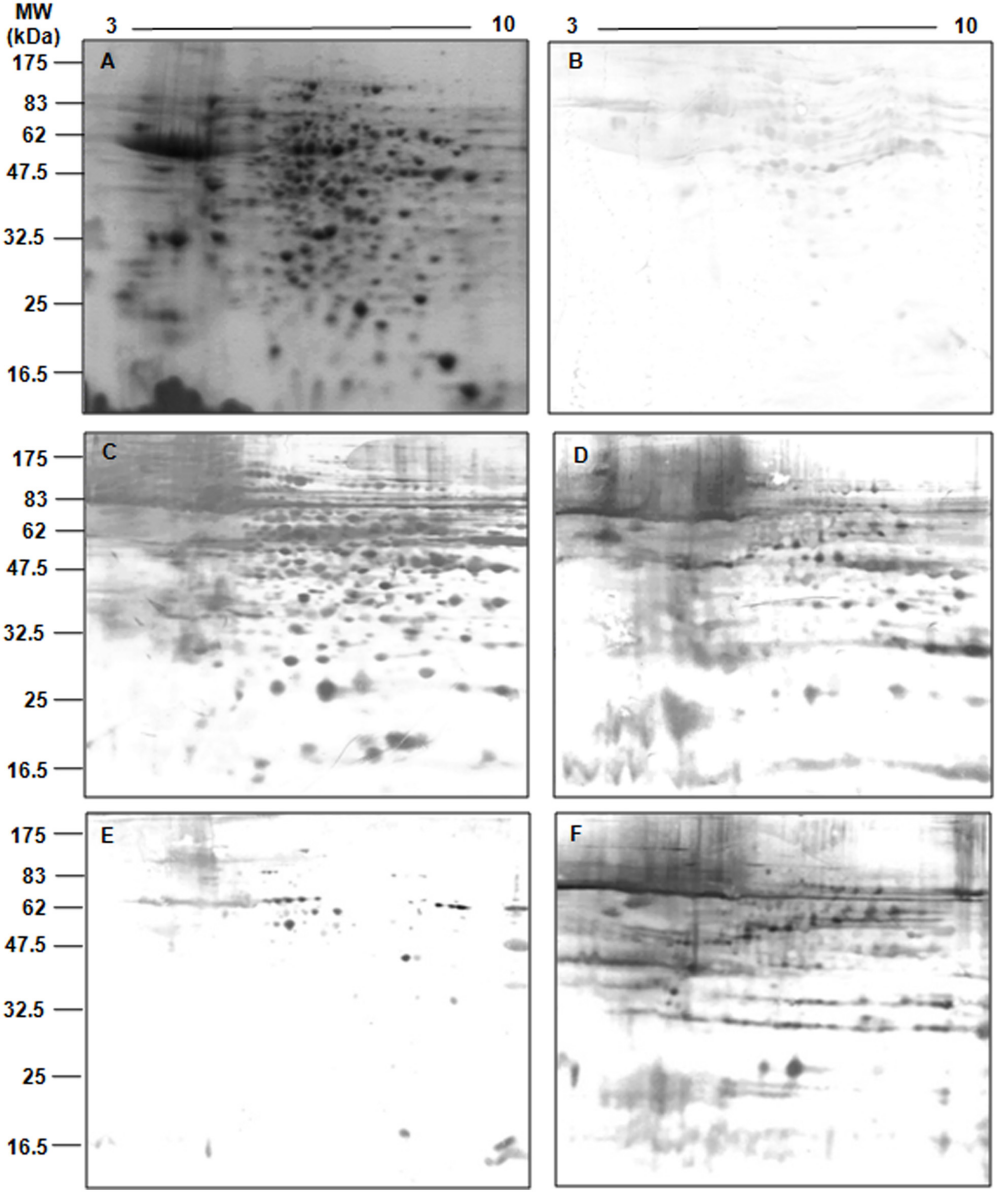
2DE and western blot analysis of serospecificity for samples from different dogs suffering from CVL. (A) Total promastigote cell extracts were separated in the first dimension with a pH gradient of 3–10 (7 cm strips) followed by 12% SDS-PAGE. The separated proteins were electroblotted onto nitrocellulose membranes and probed with different pools of sera from (B) healthy dogs (n = 5; 1:500), (C, E) asymptomatic dogs (n = 5 and n = 4, respectively; 1:500), and (D, F) symptomatic dogs (n = 3 and n = 2, respectively; 1:1,000).

**Fig 3:**
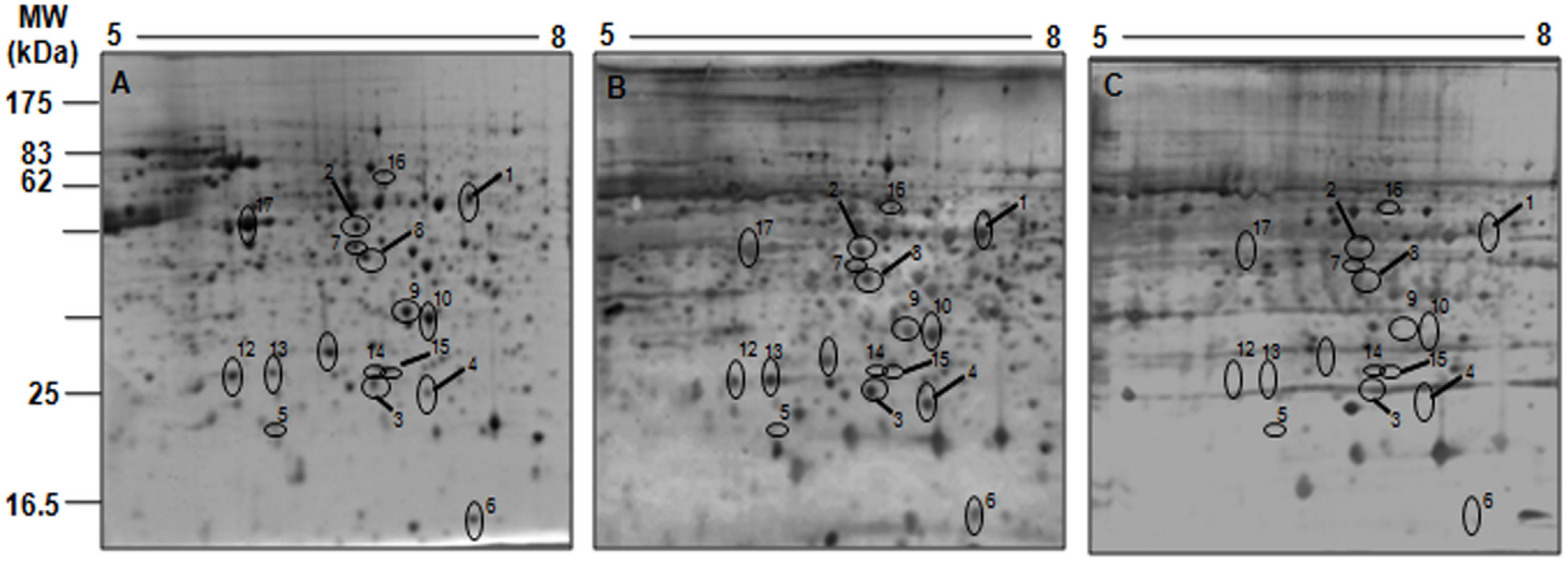
Comparison of serospecificities against total promastigote cell extracts of symptomatic (SD) and asymptomatic (AD) dogs suffering from CVL. (A) Total promastigote cell extracts were separated in the first dimension with a pH gradient of 5–8 (7-cm strips) followed by 12% SDS-PAGE. The separated proteins were electroblotted onto a nitrocellulose membrane and probed with pools of sera from (B) asymptomatic (n = 9; 1:500) and (C) symptomatic dogs (n = 5; 1:1,000). Protein spots recognized by AD sera only are numbered.

To identify the soluble proteins that were exclusively recognized by asymptomatic dog sera, pools of asymptomatic or symptomatic dog sera were used as probes in a 2-DE western blot analysis on pH range of 3 to 10. As expected, significant differences were observed in the number and pattern of antigenic spots not only between groups, but also among dogs within each group, indicating highly individualized antibody responses in dogs (Fig 2C–2F)., Interestingly, the majority of antigenic spots that were reactive to asymptomatic dog sera clustered in the acidic pH range between 4 and 9. No reactivity of these spots was observed in control blots using serum samples from healthy dogs (Fig 2). To improve antigen separation and, consequently, antigen identification, zoom gels were produced with a narrow pH range of 5 to 8, the region in 2D gels that was most densely populated with protein spots. The western blot analysis revealed approximately 671 spots after incubation with serum from asymptomatic dogs, whereas incubation with serum from symptomatic dogs resulted in 523 spots (Fig 3). Superimposition of the blots probed with sera from the two groups revealed approximately 42 spots in the 62–11 kDa range that were specifically recognized by serum from asymptomatic dogs (Fig 3B). Of these, 17 (40.4%) spots could be assigned to protein spots in CBB-stained gels (Fig 3A) and were further analyzed by LC-MS/MS for protein identification.

### Identification of *Leishmania* reactive proteins to asymptomatic dog sera

Mass spectrometry analysis of the selected spots revealed a total of 21 proteins, including 2 hypothetical and 19 known proteins, with molecular masses between 20 and 55 kDa. Reproducibility of the spot pattern in the gels and blots was very high and most of the antigens were identified in all gels tested. The difference between number of selected spots and the number of identified proteins was explained by 4 spots (spot number: 1, 2, 9, and 13) for which more than one protein was identified. The remaining 16 spots corresponded to unique proteins (Table 1). Interestingly, 4 proteins (RNA helicase, ribonucleoprotein p18, protein transport protein Sec13, and RNA-binding protein) showed considerable variation between predicted and obtained values in terms of molecular mass and isoelectric point (pI), suggesting that they underwent post-translational modifications, a major characteristic of *Leishmania* parasite proteins.

**Table 1:** Protein antigens recognized by IgG antibodies in asymptomatic dog (AD) sera.

Function	ID^a^	Protein Name	TriTrypDB accession no. *L*. *infantum*	Mr^b^ (pred/exp)	pI^c^ (pred/exp)	Pm/% Sc^d^	Score^e^
Carbohydrate metabolism	1	Dihydrolipoamide dehydrogenase	LinJ.32.3510	50.6/54.75	6.87/6.06	16/51.68	555.79
	7	Aldose-1-epimerase	LinJ.35.0990	41.5/43.41	6.24/6.48	10/46.90	168.41
	10	Prostaglandin f2-alpha synthase	LinJ.31.2210	31.7/31.9	6.65/6.87	12/65.49	385.72
	12	Phosphomannomutase	LinJ.36.2070	28.1/27.7	5.57/5.64	7/44.13	109.50
	17	Enolase	LinJ.14.1240	46.0/49.58	5.45/5.93	17/62.00	331.13
Amino acid metabolism	11	2,4-dihydroxyhept-2-ene-1,7-dioic acid aldolase	LinJ.25.2090	30.4/29.5	6.21/6.30	9/39.07	78.87
	15	Pyrroline-5-carboxylase reductase	LinJ.13.1420	28.3/27.7	6.57/6.59	2/11.4	54.97
Transcription/Translation	2	RNA helicase	LinJ.21.1820	58.8/47.50	8.62/6.16	22/53.97	294.73
	2	Eukaryotic initiation factor 4a	LinJ.01.0790	45.3/47.50	6.23/6.16	13/51.12	79.8
	4	Proteasome beta 2 subunit	LinJ.35.3880	27.6/25.62	6.61/6.34	8/29.92	158.9
	9	RNA-binding protein	LinJ.35.2240	30.2/32.5	8.13/6.74	9/67.88	155.85
Protein targeting/Signal tranduction	3	GTP-binding protein	LinJ.25.1460	24.2/26.25	6.54/6.27	9/44.44	197.25
	6	Cyclophilin 2	LinJ.06.0120	20.4/19.60	7.03/6.48	7/68.98	165.29
	8	Cyclophilin 40	LinJ.35.4830	38.5/41.36	6.00/6.52	10/42.37	128.70
	9	Activated protein kinase c receptor (LACK)	LinJ.28.2940	34.4/32.5	6.52/6.74	6/31.73	110.41
	16	Chaperonin HSP60, mitochondrial precursor	LinJ.32.1940	64.3/62.0	6.49/6.66	12/36.53	114.47
Uncharacterized proteins	5	Ribonucleoprotein p18, mitochondrial precursor	LinJ.15.0330	21.3/21.91	7.15/6.10	2/67.91	345.04
	8	Protein transport protein Sec13	LinJ.32.0050	36.3/41.36	6.02/6.52	6/33.63	52.83
	13	i/6 autoantigen-like protein	LinJ.22.1310	23.0/27.7	5.83/5.73	6/35.27	35.66
Hypothetical proteins	13	Hypothetical protein, conserved	LinJ.09.0040	22.3/27.7	5.87/5.73	5/50.48	25.02
	14	Hypothetical protein, conserved	LinJ.19.1440	25.9/28.3	6.35/6.54	10/53.51	110.44

^a^ID—The numbers correspond to the specific spots indicated in Fig 3.

^b^Mr—Molecular weight, kDa (pred, predicted; exp, experimental).

^c^pI—Isoelectric point.

^d^Pm—No. of peptides matched / % Sc (sequence coverage percentage).

^e^Score—The sum of all peptide Xcorr values above the specified score threshold (+2 ≥ 2.0, +3 ≥2.5).

Identified immunogens were functionally classified according to the GeneDB (www.genedb.org) and TriTrypDB databases (Table 1). Except for the 2 hypothetical proteins and the 3 proteins with unknown function (ribonucleoprotein p18/mitochondrial precursor, protein transport protein Sec13, and i/6 autoantigen-like protein), the immunogens identified were involved in energy metabolism, protein synthesis, and stress response. Among these proteins, eukaryotic initiation factor 4a [29,30,31], activated protein kinase c receptor (LACK) [32,33,34], and enolase [35,36] have already been characterized as vaccine candidates or Th1 immunostimulants, whereas dihydrolipoamide dehydrogenase [37], and chaperones HSP60 and cyclophilins are known therapeutic targets against leishmaniasis [38,39,40].

### Identification of T cell epitopes of immunogenic proteins through *in silico* analysis

All *Leishmania* proteins identified in the MS analysis were subjected to an *in silico* analysis to investigate the existence of potential CD4^+^ and CD8^+^ T cell epitopes. Based on a bioinformatics analysis (S2 and S3 Tables), chaperonin HSP60 was the most immunogenic protein since it had the highest MHC class I- and MHC class II-restricted overlapping epitope scores (S2 and S3 Tables), covering 58% of its amino acid sequence (Table 2). Dihydrolipoamide dehydrogenase, RNA helicase, and one hypothetical protein (LinJ.09.0040) also contained multiple high-scoring epitopes, covering approximately 50% of their amino acid sequences (S2 and S3 Tables and Table 2). Cyclophilin 2 could also be considered an immunogenic protein since the predicted CD4^+^ and CD8^+^ T cell epitopes spanned the majority of its sequence (75.4%), despite low scores (S2 and S3 Tables). Furthermore, enolase and cyclophilin 40 contained multiple MHC class II-restricted epitopes, as shown in S3 Table. It must be noted that the majority of extracted peptides were recognized by two different HLA alleles for the two algorithms. Combined analysis with ANTIGENpro and VaxiJen servers also indicated that dihydrolipoamide dehydrogenase, aldose-1-epimerase, cyclophilin 2, cyclophilin 40, RNA helicase, LACK, chaperonin HSP60, and the two hypothetical proteins are highly antigenic molecules (Table 3). Combined, the above data regarding highly scored HLA binders and antigenicity values indicated that chaperonin HSP60, hypothetical protein LinJ.09.0040, dihydrolipoamide dehydrogenase, enolase, cyclophilin 2, and cyclophilin 40 are potential vaccine candidates that deserve further investigation (Table 4).

**Table 2:** Percentage (%) of amino acid sequence coverage of identified *L*. *infantum* proteins based on their content in high binding MHC class I and/or II-restricted peptides.

ID^a^	Protein Name	Protein length (aa)	No. of MHC class I peptides binding to ≥ 2 HLA^b^	No. of MHC class II peptides with score ≤ 10 nM^c^	% aa sequence coverage^d^
6	Cyclophilin 2	187	4	2	75.4
8	Protein transport protein Sec13	254	-	8	65.3
16	Chaperonin HSP60, mitochondrial precursor	594	6	11	58
11	2,4-dihydroxyhept-2-ene-1,7-dioic acid aldolase	279	-	9	57.7
2	RNA helicase	517	2	6	50.9
1	Dihydrolipoamide dehydrogenase	476	3	8	50
13	Hypothetical protein, conserved	619	2	11	49.9
3	GTP-binding protein	216	-	4	49
10	Prostaglandin f2-alpha synthase	284	2	4	48.6
2	Eukaryotic initiation factor 4a	403	3	6	46.7
4	Proteasome beta 2 subunit	254	2	9	46.5
5	Ribonucleoprotein p18, mitochondrial precursor	187	-	3	43.9
17	Enolase	429	-	12	43
13	i/6 autoantigen-like protein	207	-	2	43
8	Cyclophilin 40	354	-	11	39.5
9	RNA-binding protein	274	3	12	38.3
7	Aldose-1-epimerase	371	3	3	38
14	Hypothetical protein, conserved	228	-	3	37.7
9	Activated protein kinase c receptor (LACK)	312	-	-	32
12	Phosphomannomutase	247	-	7	28.7
15	Pyrroline-5-carboxylase reductase	272	1	5	25

^a^ID—The numbers correspond to the specific spots indicated in Fig 3.

^b^MHC class I-restricted peptides were chosen based on their ability to bind with high affinity (NetCTL: ≥0.75, SYFPEITHI: ≥20) to more than two HLA types according to both NetCTL and SYFPEITHI algorithms.

^c^MHC class II-restricted peptides were chosen based on their ability to bind with high affinity (NetMHCII: ≤10nM) to the HLA-DRB1 allele.

^d^Percentage (%) of amino acid sequence coverage from the high binding MHC class I and/or II-restricted peptides.

**Table 3:** *In silico* evaluation of the identified proteins antigenicity.

ID	Protein Name	ANTIGENpro value	VaxiJen value
**1**	**Dihydrolipoamide dehydrogenase**^a^	**0.680**	**0.5188**
**2**	**RNA helicase**^a^	**0.807**	**0.6224**
2	Eukaryotic initiation factor 4a	0.363	0.4589
3	GTP-binding protein	0.845	0.4285
**4**	**Proteasome beta 2 subunit**^a^	**0.667**	**0.5354**
5	Ribonucleoprotein p18, mitochondrial precursor	0.916	0.4645
**6**	**Cyclophilin 2**^a^	**0.801**	**0.6418**
**7**	**Aldose-1-epimerase**^a^	**0.940**	**0.5573**
**8**	**Cyclophilin 40**^a^	**0.934**	**0.7018**
8	Protein transport protein Sec13	0.706	0.4934
**9**	**RNA-binding protein**^a^	**0.840**	**0.5624**
**9**	**Activated protein kinase c receptor (LACK)**^a^	**0.809**	**0.6195**
10	Prostaglandin f2-alpha synthase	0.731	0.1847
11	2,4-dihydroxyhept-2-ene-1,7-dioic acid aldolase	0.600	0.4964
12	Phosphomannomutase	0.477	0.4512
**13**	**Hypothetical protein**^a^	**0.517**	**0.5354**
13	I6 autoantigen-like protein	0.703	0.3822
**14**	**Hypothetical protein**^a^	**0.606**	**0.5616**
15	Pyrroline-5-carboxylase reductase	0.400	0.5467
**16**	**Chaperonin HSP60, mitochondrial precursor**^a^	**0.650**	**0.5073**
17	Enolase	0.599	0.4598

^a^Bold indicates proteins with scores > 0.5 using both algorithms.

**Table 4:** Proteins selected on the basis of predicted antigenicity and their content on highly scored MHC class I- and MHC class II-restricted epitopes.

ID	Protein Name^b^	No. of MHC class Iepitopes^a^^,^^c^	No. of MHC class IIepitopes^a^	Antigenicity values (ANTIGENpro/VaxiJen)
16	Chaperonin hsp60, mitochondrial precursor	6	11	0.650/0.5073
3	Hypothetical protein LinJ.09.0040	2	11	0.517/0.5354
1	Dihydrolipoamide dehydrogenase	3	8	0.680/0.5188
6	Cyclophilin 2	4	2	0.801/0.6418
17	Enolase	-	12	0.599/0.4598
8	Cyclophilin 40	-	11	0.934/0.7018

^a^NetCTL and NetMHCII algorithms were used to identify and select the highly scored MHC class I- and MHC classII-restricted epitopes, respectively.

^b^The proteins were selected based on the number of peptides predicted to bind with high affinity to multiple HLAs.

^c^MHC class I-resticted peptides were chosen based on their ability to bind with high affinity to more than two HLA types.

## Discussion

In the present study, combined immunoproteomic and bioinformatic approaches were applied in order to identify immunoreactive to asymptomatic CVL serum proteins from *L*. *infantum* parasites total extracts with potential use in the development of prophylactic vaccines or sophisticated diagnostic tools. For this purpose, total extracts obtained from late-log promastigotes were selected because they are commonly used in the sero-diagnosis and previous studies showed their high content on Th1-stimulatory molecules [36,41,42,43].

Until now, efforts to identify immunogenic proteins through immunoproteomics by using sera of asymptomatic and/or symptomatic humans or dogs have focused on the use of *L*. *chagasi* or *L*. *donovani* parasites, etiological agents of ZVL in South America and anthroponotic VL in India, respectively [15,44,45,46]. The current study offers complementary findings, since it is the first to investigate the immunoreactive proteome of *L*. *infantum* parasite associated with asymptomatic CVL in the endemic Meditteranean area.

In asymptomatic CVL, humoral responses although not correlated directly with protection, are largely T-cell dependent [28,46]. Consequently, antigens that react with antibodies in asymptomatic animal sera may be associated with protective immune responses and may represent potential vaccine candidates. In agreement with previous findings, in the present study, asymptomatic CVL sera was characterized by different IgG isotype reactivity pattern compared to that detected when using symptomatic dog sera, which might be indicative of immunity and protection against reinfection. It must be noted that asymptomatic animals participating in the study did not developed any symptoms of the disease for 2 years following serum collection and according to previous studies [12,14], asymptomatic dogs with the above profile are considered as resistant to *Leishmania* infection. For the identification of the immunoreactive parasite antigens, a high resolution 2-DE gel map of total promastigote extract was obtained. Surprisingly, despite low IgG content, asymptomatic dogs’ sera detected more immunoreactive spots than those detected when using sera obtained from symptomatic dogs. Previous proteomic analysis studies have shown a reduction in serum reactivity against most of the parasite proteins in the case of asymptomatic disease compared to active disease [15, 47]. This discrepancy could be attributed to the fact that proteome analysis was conducted to different pH ranges. In contrast to the present study where pH range of 5 to 8 was applied, previous studies analyzed parasite proteome in the pH range of 4 to 7. It was observed that selection of specific narrow pI range may have limitations in analysis and consequently give different results. For example, Brotherton et al. reported several highly basic proteins in promastigote and amastigote protein extracts with free-flow electrophoresis, which were not previously identified in the acidic pH range of 4 to 7 [48]. Moreover, overall differences may be attributed to differences in the antigenicity of the parasites used. Until now, comparative immunoproteomic studies as far as concerning VL were conducted with *L*. *chagasi* [15,46], the etiological agent of VL in South America, whereas in the present study the proteome of *L*. *infantum* which is endemic in Mediterranean basin was analyzed. Differences in the habitat of parasite may result in differences in parasite’s antigenicity and subsequently virulence. It is of great importance to point out that despite the fact that *L*. *chagasi* and *L*. *infantum* are considered the same species, infection with *L*. *chagasi* leads to much higher number of deaths in Brazil than in Europe. The differences detected in immunoproteome between symptomatic and asymptomatic dog sera seem to indicate antigenicity drifts which do not correlate necessarily with major differences in the proteins expressed, as detected in 2-DE gels in all cases. Loss of antigenicity, as also indicated by the present study, suggests immune evasion by the parasites and is related primarily to the persistence of the infections in the individual host. Such responses to the hosts’ immune responses have been also described for other protozoans, such as plasmodia [49,50] and trypanosomes [51,52], and related primarily to the persistence of the infections in the individual host [53].

The comparative analysis revealed 42 protein spots that were specifically recognized by components of asymptomatic dog sera. Of these, only 17 proteins spots representing 21 proteins were successfully identified using MS. It was suggested that anti-*Leishmania* antibody responses are preliminary directed against highly conserved antigens that are typically parts of multi-component complexes, such as heat-shock proteins, ribosomal proteins or proteins of the DNA replication and transcription that are essential for infectivity, and therefore survival, in parasites [54]. Indeed, most of the proteins identified fall into five major categories according to their predicted function: (i) carbohydrate metabolism, such as enolase, (ii) amino acid metabolism such as 2,4-dihydroxyhept-2-ene-1,7-dioic acid aldolase, (iii) protein synthesis such as eukaryotic initiation factor 4a, (iv) signal transduction such as LACK, and (v) stress response such as cyclophilins and chaperonin HSP60. Additionally, the identified proteins included 2 hypothetical conserved proteins and 3 proteins with unknown function (ribonucleoprotein p18, protein transport protein Sec13, and i/6 autoantigen-like protein). As expected, some of the proteins identified in the present work have been previously associated with humoral responses in VL and tegumentary leishmaniasis (TL) and are considered important candidate antigens for diagnosis, such as enolase [55], chaperonin HSP60 [56] and LACK [57,58,59]. Interestingly, enolase, LACK, cyclophilin, eukaryotic intiation factor 4a and chaperonin HSP60, have been identified in the secretome of different *Leishmania* parasites, further enhancing their use in diagnostic applications or as potential immunoprophylactic antigens [60,61,62]. Their presence in secretome was attributed to an unconventional bacterial secretion pathway present in *Leishmania*, as shown by Cuervo et al [62]. It is worth emphasizing that the commercially available vaccine against CVL in Europe, CaniLeish^®^, is consisted of excreted/secreted *L*. *infantum* promastigote antigens [63]. Until now, only promastigote surface antigen (PSA) was identified as the major component of this fraction [64]. However, a main argument for an anti-promastigote vaccine is that protective immunity is disrupted by sand fly’s saliva immunomodulators. Since amastigote form is the stage of parasite that survives inside mammalian host and is responsible for the development of active disease, researchers have been focused on the identification of immunoreactive proteins in amastigote proteome [10,65]. Interestingly, the immunodominant antigens to asymptomatic dog sera, enolase [15,55] and chaperonin HSP60 [56, 66] were found to be abundantly expressed also in the amastigote form of the parasite. Subsequently, verification of these proteins in *L*. *infantum* amastigote lysate as immunoreactive antigens to asymptomatic dog sera would enhance further their use as vaccine candidates.

In the field of vaccine development against CVL, researchers still face challenges with respect to the heterogeneity in immunological profiles among dogs. HLA molecules are highly polymorphic, and an effective vaccine should contain MHC class I- and/or II-restricted epitopes that have the ability to bind to the most frequent MHC in the dog or human population. Accordingly, a recent review supports the combined application of immunoproteomics with bioinformatics to address this problem, i.e., to identify several antigens containing multiple promiscuous MHC class I- and/or II-binding epitopes [67]. Experimental validation of molecules identified by the above approach has yielded encouraging results in terms of IFN-γ production and protective efficacy against different pathogens, such as *Coccidioides posadasii* [68,69]. In the case of CVL, Costa et al. revealed 5 candidate vaccine proteins, i.e., phosphomannomutase, prostaglandin f2-alpha synthase, elongation factor 1a, alpha tubulin, and one hypothetical protein, with high CD8^+^ T cell epitope contents, using the combined application of immunoproteomics and bioinformatics [44]. In the present study, a bioinformatics analysis of detected molecules was used to identify 7 potential vaccine candidates. These proteins were chaperonin HSP60, cyclophilin 2, enolase, cyclophilin 40, dihydrolipoamide dehydrogenase, RNA helicase, and one hypothetical protein (LinJ.09.0040), and they contained multiple promiscuous MHCI- and/or MHCII-restricted T cell epitopes against the most frequent HLA alleles in the human population (since algorithms have not been developed for canine MHC molecules). The discrepancy between the proteins identified in the present study and those identified by Costa et al. may be due to the use of parasites (*L*. *infantum* versus *L*. *chagasi*) isolated from different geographical regions.

According to the present findings, chaperones HSP60, cyclophilin 4, and cyclophilin 40 were the most highly immunogenic molecules in terms of MHC class I- and MHC class II-restricted epitope content. In general, chaperones comprise several highly conserved families of protein folding facilitators that play important roles in responses to chemical and physiological stresses. Furthermore, they are related to parasite viability and resistance against treatment [40,70,71,72]. They were, also, found to play important role in humoral immune response against various infectious diseases and autoimmune responses. Requena et al. have characterized heat shock proteins, among others, as pan-antigens [54,73]. Indeed, a previous study has shown that *L*. *major* HSP60 is recognized specifically by leishmaniasis patients and the antibodies raised showed no reactivity against human HSP60 due to low sequence similarity [56]. Furthermore, mice vaccination with cyclophilin protein 1 confers high protection against *L*. *infantum* infection by generating specific CD4^+^ and CD8^+^ effector and memory T cells [74].

Enolase is considered an important molecule for parasite survival owing to its role in energy production and more specifically in the processes of glycolysis and glyconeogenesis. It also contributes to parasite infectivity, as evidenced by its abundant expression in antimony- and amphotericin B-resistant *Leishmania* parasites [38,75,76]. Previous studies of *Plasmodium* spp. [77], *Candida albicans* [78,79], *Chlamydia pneumonia* [80], and *Streptococcus* spp. [81,82] have reported that enolase is an important candidate molecule for vaccine development. A proteomics analysis of *L*. *donovani*, the etiological agent of human VL, identified enolase in a cytoplasmic sub-fraction ranging from 89.9 to 97.1 kDa that was able to elicit significant Th1 responses in PBMCs and lymphocytes obtained from cured *Leishmania* patients and hamsters, respectively [36,41]. In another comparative study between humans with active VL and resistant or successfully treated humans, enolase showed increased reactivity with sera from asymptomatic individuals, strengthening the idea that some antigens, such as enolase, are specifically associated with protection against VL [46]. In an experimental VL model have shown that enolase has prophylactic potential, since hamster vaccination results in a decrease of about 90% in the parasitic load and significant upregulation of Th1-signature molecules, such as iNOS, IFN-γ, TNF-α, and IL-12 [35]. In the same study, an *in silico* analysis of the enolase amino acid sequence revealed several promiscuous CD4^+^ and CD8^+^ T-cell epitopes. Our *in silico* analysis showed that enolase contains several promiscuous CD4^+^ T cell epitopes, supporting the idea that it is a prophylactic molecule against leishmaniasis that is capable of inducing Th1 immune responses.

Little is known about the immunogenicity of dihydrolipoamide dehydrogenase, except that it is included in the same sub-fraction of *L*. *donovani* as enolase, which induces strong Th1 immune responses by PBMCs [41]. However, it is classified among the molecules that play a role in *L*. *infantum* infectivity and resistance against amphotericin B [76]. Furthermore, no detailed information about the biological function of RNA helicase is available to date. Characterization of this protein as well as the identified hypothetical protein are essential, as they could prove to be significant vaccine candidates against leishmaniasis.

It is noteworthy that among the proteins identified in the immunoproteomic analysis, two are known for their immunostimulatory potential (eukaryotic initiation factor 4a (elF-4a) and LACK) and induce high levels of IL-12 and TNFα production in antigen-presenting cells [29,30,82,83,84,85,86]. These proteins contained a low number of potential MHC class I- and/or II-restricted epitopes according to *in silico* analysis compared to the other proteins identified. Previous studies have shown that mouse vaccinations with either protein alone fail to elicit long-term protective immune responses against different *Leishmania* species [32,87,88]. In contrast, these proteins confer protection only when used in combination with other antigens, as in the case of recombinant tri-fusion vaccines (leish111; leish110f) for elF-4a [31,88] or as DNA constructs as in the case of LACK [33,34].

In summary, in this study, the combined application of immunoproteomics and bioinformatics enabled the identification of immunogens containing MHC class I- and MHC class II-restricted epitopes on the soluble extract of *L*. *infantum*, the etiological agent of VL in the Mediterranean basin. Most of them have been associated with physiological and virulence functions of this parasite. However, further studies are required to unveil their expression and immunodominace in amastigote lysates by using a larger dog serological panel, and subsequently their protective efficacy for the development of an effective vaccine against CVL.

## Supporting Information

S1 Fig Characterization of IgG reaction patterns associated with resistance or susceptibility. Total promastigote cell extract was separated with 12% SDS-PAGE and electrotransferred to a nitrocellulose membrane. The membranes were probed with asymptomatic dog sera (lanes 1–6) or symptomatic dog sera (lanes 7–11) at a 1:500 and 1:1000 dilutions.
(TIF)

S1 Table Classification of dogs participating in the study. Age, sex, serum code IFAT titer, OD value and IgG2/IgG1 ratio obtained from ELISA, and qPCR results for each dog enrolled in the immunoproteomics study are shown.
(DOCX)

S2 Table *In silico* predicted *L*. *infantum* MHC class I-restricted high binding 9-mer peptides. Promiscuous 9-mer peptides specific to human MHC class I molecules after a combined *in silico* analysis with the SYFPEITHI and NetCTL algorithms.
(DOCX)

S3 Table *In silico* predicted *L*. *infantum* MHC class II-restricted high binding 15-mer peptides. Promiscuous 15-mer peptides specific to human MHC class II molecules after a combined *in silico* analysis with the SYFPEITHI and NetMHCII algorithms.
(DOCX)
