# Supplementary figures and images for: Identification of Immunoreactive Leishmania infantum Protein Antigens to Asymptomatic Dog Sera through Combined Immunoproteomics and Bioinformatics Analysis
Source: PLoS One. 2016 Feb 23;11(2):e0149894. doi: 10.1371/journal.pone.0149894 (PMC4764335; doi:10.1371/journal.pone.0149894)

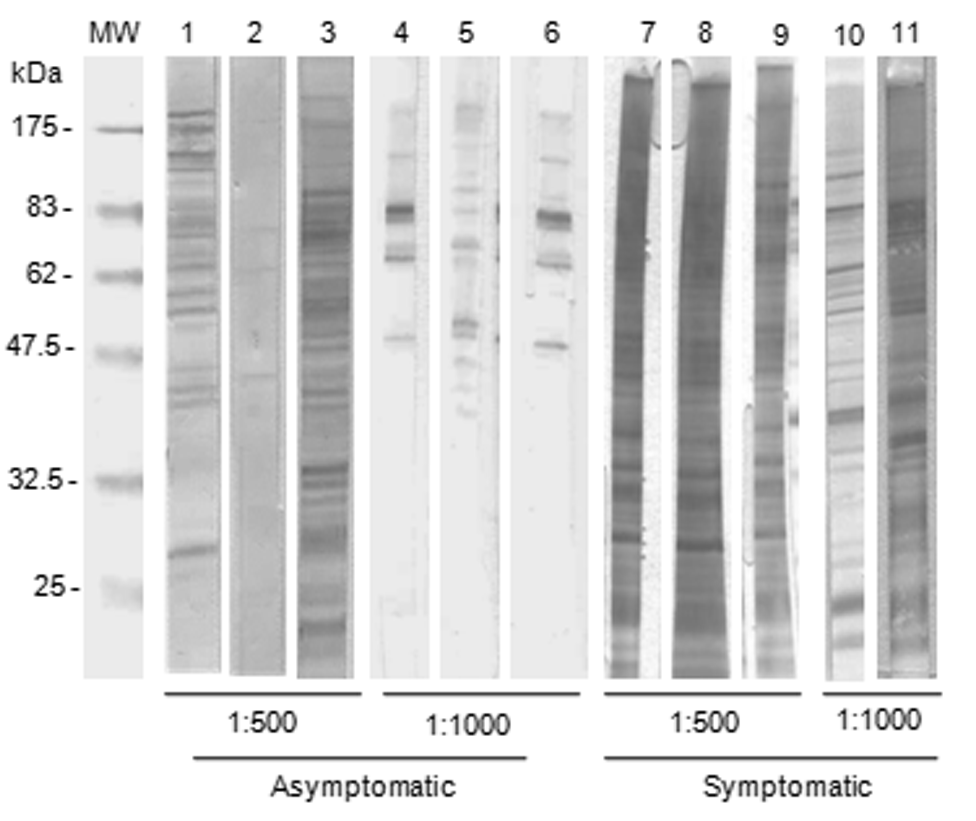

Supplement: S1 Fig — Total promastigote cell extract was separated with 12% SDS-PAGE and electrotransferred to a nitrocellulose membrane. The membranes were probed with asymptomatic dog sera (lanes 1–6) or symptomatic dog sera (lanes 7–11) at a 1:500 and 1:1000 dilutions. (TIF) [file pone.0149894.s001.tif]
