# Supplementary material for: Identification of Immunoreactive Leishmania infantum Protein Antigens to Asymptomatic Dog Sera through Combined Immunoproteomics and Bioinformatics Analysis
Source: PLoS One. 2016 Feb 23;11(2):e0149894. doi: 10.1371/journal.pone.0149894 (PMC4764335; doi:10.1371/journal.pone.0149894)
Supplement: S1 Table — Age, sex, serum code IFAT titer, OD value and IgG2/IgG1 ratio obtained from ELISA, and qPCR results for each dog enrolled in the immunoproteomics study are shown. (DOCX) [file pone.0149894.s002.docx]

**S1Table. Classification of dogs participating in the study.**

| **a/a** | **Sample Code** | **Sex** | **Age (years)** | **Race** | **IFAT**  **(titer)^1^** | **ELISA**  **(OD)^2^** | **IgG2/IgG1**  **(OD_IgG2_/OD_IgG1_)^3^** | **PCR** | |
| --- | --- | --- | --- | --- | --- | --- | --- | --- | --- |
|  |  |  |  |  |  |  |  | **blood** | **swab** |
| **Asymptomatic Dogs** | | | | | | | | | |
| 1 | 222361 | female | 2 | German Shepherd | 1/100 | 0.537 | 1.7 (0.168/0.106) | - | ND |
| 2 | 222478 | male | 7 | mongrel | 1/100 | 0.340 | 1.6 (0.279/0.052) | - | + |
| 3 | 222626 | male | 8 | Golden Retriever | 1/100 | 0.396 | 6.5 (0.355/0.055) | + | ND |
| 4 | 162037 | female | 6 | Golden Retriever | 1/100 | 0.255 | 11.1 (0.435/0.016) | - | - |
| 5 | 150978 | male | 7 | Griffon | 1/100 | 0.374 | 10.1 (0.221/0.022) | - | - |
| 6 | 161001 | female | ▬ | Boxer Pitbull | 1/100 | 0.461 | 20.7 (0.620/0.030) | + | - |
| 7 | 224068 | male | 7 | Labrador Retriever | 1/100 | 0.374 | 4.2 (0.113/0.027) | - | + |
| 8 | 224308 | male | 2 | mongrel | 1/100 | 0.392 | 5.2 (0.303/0.058) | - | + |
| 9 | 127769 | female | 1 | German Shepherd | 1/100 | 0.347 | 13.2 (0.403/0.032) | - | + |
| **Symptomatic Dogs** | | | | | | | | | |
| 10 | 223118 | male | ▬ | Kurzhaar | >1/1600 | 1.844 | 3.1 (2.079/0.675) | + | - |
| 11 | 150988 | female | 11 | mongrel | >1/1600 | 2.029 | 1.7 (2.219/1.333) | + | + |
| 12 | 189169 | male | 10 | Golden Retriever | 1/1600 | 1.313 | 4.7 (1.357/0.287) | + | - |
| 13 | 161021 | female | 2.5 | mongrel | 1/1600 | 1.990 | 3.2 (2.088/0.661) | + | - |
| 14 | 150998 | ▬ | 9 | mongrel | 1/1600 | 1.822 | 4.1 (2.018/0.487) | - | + |
| ^1^IFAT titer < 1/200: Asymptomatic Dogs, >1/200: Symptomatic Dogs  ^2^ELISA cut-off value 0.334 using sera from healthy dogs at dilution diluted 1/400  ^3^IgG2/IgG1 ratio at 1/400 dilution | | | | | | | | | |
