# Supplementary material for: Identification of Immunoreactive Leishmania infantum Protein Antigens to Asymptomatic Dog Sera through Combined Immunoproteomics and Bioinformatics Analysis
Source: PLoS One. 2016 Feb 23;11(2):e0149894. doi: 10.1371/journal.pone.0149894 (PMC4764335; doi:10.1371/journal.pone.0149894)
Supplement: S2 Table — Promiscuous 9-mer peptides specific to human MHC class I molecules after a combined in silico analysis with the SYFPEITHI and NetCTL algorithms. (DOCX) [file pone.0149894.s003.docx]

**S2 Table. *In silico* predicted *L. infantum* MHC class I-restricted high binding 9-mer peptides.**

| **ID^a^** | **Protein Name** | **Amino acid position of peptide sequence^a^** | **HLA supertype** | |
| --- | --- | --- | --- | --- |
|  |  |  | **NetCTL** | **SYFPEITHI** |
| 16 | Chaperonin hsp60, mitochondrial precursor | 214-SPYFVTNTK-222 | A3 , B7 | A3 |
|  |  | 242-SVAHILPAL-250 | A2, B7 | A2 |
|  |  | 379-RLVERLAKL-387 | A2 | A2 , A3 |
|  |  | 386-KLSGGVAVI-394 | A2 | A2, A3 |
|  |  | 414-ALNATRAAV-422 | A2 | A2 , A3 |
|  |  | 426-ILAGGGTGL-434 | A2 | A2 , A3 |
| 6 | Cyclophilin 2 | 2-RVVAVLAVV-10 | A2, B7 | A2 , A3 |
|  |  | 3-VVAVLAVVL-11 | B7 | A2 , A3 |
|  |  | 153-KVLDGMDVV-161 | A2 | A2 , A3 |
|  |  | 154-VLDGMDVVL-162 | A2 | A2, A3 |
| 1 | Dihydrolipoamide dehydrogenase | 60-ALLHATHLY-68 | A3 | A3 , B62 |
|  |  | 204-RLGAEVTVV-212 | A2 | A2 , A3 |
|  |  | 431-MIAEPTLAM-439 | A2, B7 | A2 |
| 2 | Eukaryotic initiation factor 4a | 36-NLLRGIYSY-44 | A3 | A2 , B62 |
|  |  | 143-KLQAGVIVA-151 | A2 | A2 , A3 |
|  |  | 378-LLHEIEAHY-386 | A3 | A3 , B62 |
| 7 | Aldose-1-epimerase | 78-RVAGGVFTL-86 | A2 | A2 , A3 |
|  |  | 117-KLIETANVI-125 | A2 | A2 , A3 |
|  |  | 123-NVIGVRFNY-131 | A3 | A3, B62 |
| 9 | RNA-binding protein | 130-GQYTAGGYY-138 | A3 | B62 |
|  |  | 196-KLFVSNLPF-204 | A3 | A3, B62 |
|  |  | 210-ALRETFQQV-218 | A2 | A2, A3 |
| 2 | RNA helicase | 335-TLHGLAQFY-343 | A3 | A3 , B62 |
|  |  | 354-RLAEILDVV-362 | A2 | A2 , A3 |
| 3 | Hypothetical protein, conserved | 96-SLYEFGLSV-104 | A2 | A2 , A3 |
|  |  | 173-MLTSLAATV-181 | A2 | A2 , A3 |
| 4 | Proteasome beta 2 subunit | 104-RVLEALTLL-112 | A2 , B7 | A2 |
|  |  | 111-LLKRHLYRY-119 | A3 | A3 , B62 |
| 10 | Prostaglandin f2-alpha synthase | 79-KLWNTEQGY-87 | A3 | A3, B62 |
|  |  | 210-ILAAIGAKY-218 | A3 | A3 , B62 |
| 15 | Pyrroline-5-carboxylase reductase | 117-RVMPNIPSF-125 | A24, B7 | B62 |

^a^ID – The numbers correspond to the specific spots indicated in Fig 2.

^b^MHC class I-restricted peptides were chosen based on their ability to bind with high affinity (NetCTL: ≥0.75, SYFPEITHI: ≥20) to more than two HLA types according to both NetCTL and SYFPEITHI algorithms.
