# Supplementary material for: Identification of Immunoreactive Leishmania infantum Protein Antigens to Asymptomatic Dog Sera through Combined Immunoproteomics and Bioinformatics Analysis
Source: PLoS One. 2016 Feb 23;11(2):e0149894. doi: 10.1371/journal.pone.0149894 (PMC4764335; doi:10.1371/journal.pone.0149894)
Supplement: S3 Table — Promiscuous 15-mer peptides specific to human MHC class II molecules after a combined in silico analysis with the SYFPEITHI and NetMHCII algorithms. (DOCX) [file pone.0149894.s004.docx]

**S3 Table. *In silico* predicted *L. infantum* MHC class II-restricted high binding 15-mer peptides.**

| **ID^a^** | **Protein Name** | **Amino acid position of peptide sequence^b^** | **HLA-DRB1*0101** | |
| --- | --- | --- | --- | --- |
|  |  |  | **NetMHCII** | **SYFPEITHI** |
| 16 | Chaperonin hsp60, mitochondrial precursor | 17-KDIRYGMEARNALLA-31 | 26.5 | 24 |
|  |  | 20-RYGMEARNALLAGVE-34 | 41.7 | 24 |
|  |  | 31-AGVENLVKAVGVTLG-45 | 23.6 | 26 |
|  |  | 33-VENLVKAVGVTLGPK-47 | 37.3 | 27 |
|  |  | 47-KGRNVILEMPYASPK-61 | 28.0 | 21 |
|  |  | 48-GRNVILEMPYASPKI-62 | 48.3 | 23 |
|  |  | 76-EDSFENLGANLVRQV-90 | 3.9 | 35 |
|  |  | 83-GANLVRQVAGLTNDN-97 | 13.0 | 23 |
|  |  | 84-ANLVRQVAGLTNDNA-98 | 19.8 | 24 |
|  |  | 137-CREVLISLAEQSRPV-151 | 33.4 | 27 |
|  |  | 154-KSEITQVAMISANMD-168 | 39.8 | 23 |
|  |  | 156-EITQVAMISANMDQE-170 | 21.0 | 21 |
|  |  | 157-ITQVAMISANMDQEI-171 | 15.7 | 23 |
|  |  | 229-ENALVYVANRKLTSV-243 | 9.3 | 25 |
|  |  | 240-LTSVAHILPALNYAI-254 | 18.5 | 20 |
|  |  | 249-ALNYAIQQKRPLLVI-263 | 10.1 | 28 |
|  |  | 251-NYAIQQKRPLLVIAE-265 | 15.2 | 22 |
|  |  | 274-TFLYNKIQGRISGCA-288 | 4.9 | 36 |
|  |  | 277-YNKIQGRISGCAVKA-291 | 17.4 | 22 |
|  |  | 294-FGDMRINQLQDIAVF-308 | 46.4 | 21 |
|  |  | 296-DMRINQLQDIAVFTG-310 | 16.7 | 25 |
|  |  | 302-LQDIAVFTGSQMISE-316 | 42.5 | 24 |
|  |  | 342-RDECILMEGGGSAIA-356 | 24.1 | 26 |
|  |  | 343-DECILMEGGGSAIAV-357 | 29.0 | 26 |
|  |  | 344-ECILMEGGGSAIAVE-358 | 26.3 | 25 |
|  |  | 345-CILMEGGGSAIAVEE-359 | 37.8 | 23 |
|  |  | 381-VERLAKLSGGVAVIK-395 | 6.0 | 34 |
|  |  | 388-SGGVAVIKVGGASEV-402 | 48.2 | 24 |
|  |  | 391-VAVIKVGGASEVEIN-405 | 47.5 | 26 |
|  |  | 406-EKKDRIIDALNATRA-420 | 9.7 | 27 |
|  |  | 409-DRIIDALNATRAAVS-423 | 4.3 | 33 |
|  |  | 412-IDALNATRAAVSEGI-426 | 6.9 | 25 |
|  |  | 421-AVSEGILAGGGTGLL-435 | 24.7 | 21 |
|  |  | 423-SEGILAGGGTGLLMA-437 | 8.9 | 28 |
|  |  | 424- SEGILAGGGTGLLMA-428 | 13.6 | 23 |
|  |  | 430-GGTGLLMASLRLESI-444 | 43.3 | 21 |
|  |  | 433-GLLMASLRLESISKD-447 | 49.3 | 24 |
|  |  | 458-VNIVKKAIGLPARYI-472 | 7.5 | 27 |
|  |  | 461-VKKAIGLPARYIANN-475 | 10.9 | 25 |
|  |  | 494-SFGYNAQTGEYVNMF-498 | 49.8 | 33 |
|  |  | 501-TGEYVNMFEAGIIDP-515 | 7.3 | 27 |
|  |  | 502-GEYVNMFEAGIIDPM-516 | 17.1 | 27 |
|  |  | 510-AGIIDPMKVVKSAVV-524 | 26.5 | 23 |
|  |  | 513-IDPMKVVKSAVVNAC-527 | 28.9 | 29 |
|  |  | 518-VVKSAVVNACSVAGM-532 | 13.4 | 22 |
|  |  | 528-SVAGMMITTEAAVVE-542 | 36.0 | 21 |
|  |  | 530-AGMMITTEAAVVEKD-544 | 30.0 | 25 |
| 3 | Hypothetical protein LinJ.09.0040 | 3-AKEFVQRFPKSALSA-17 | 8.00 | 26 |
|  |  | 9-RFPKSALSAKLVLSL-23 | 1.10 | 25 |
|  |  | 17-AKLVLSLESTEQRDF-31 | 50.0 | 24 |
|  |  | 56-NVAITEDVALAAVQA-60 | 32.0 | 25 |
|  |  | 59-ITEDVALAAVQAVGL-63 | 25.1 | 25 |
|  |  | 62-DVALAAVQAVGLDPL-76 | 7.5 | 32 |
|  |  | 67-AVQAVGLDPLSAPLW-81 | 11.9 | 23 |
|  |  | 95-KSLYEFGLSVPLHGW-109 | 39.5 | 25 |
|  |  | 163-RQAWLDLFSCMLTSL-177 | 4.8 | 32 |
|  |  | 167-LDLFSCMLTSLAATV-181 | 28.0 | 30 |
|  |  | 168-DLFSCMLTSLAATVI-182 | 5.6 | 23 |
|  |  | 170-FSCMLTSLAATVIAR-184 | 5.7 | 24 |
|  |  | 171-SCMLTSLAATVIARD-185 | 5.5 | 34 |
|  |  | 206-DSCWYQLALFQLRIL-220 | 7.1 | 26 |
|  |  | 214-LFQLRILEDAEAARK-228 | 14.0 | 30 |
|  |  | 231-ASGIAAAGPSFALEN-245 | 19.2 | 23 |
|  |  | 286-LDGVTKDRLRALRSV-300 | 22.6 | 20 |
|  |  | 288-GVTKDRLRALRSVGK-302 | 29.4 | 24 |
|  |  | 291-KDRLRALRSVGKAAA-305 | 10.9 | 26 |
|  |  | 294-LRALRSVGKAAAQQG-308 | 7.4 | 25 |
|  |  | 346-SPTDALLLGSEAAQY-360 | 28.7 | 25 |
|  |  | 347-PTDALLLGSEAAQYH-361 | 23.9 | 24 |
|  |  | 385-HHRGKIMASWNSLVR-399 | 31.1 | 24 |
|  |  | 391-MASWNSLVRIESLLG-405 | 10.2 | 27 |
|  |  | 394-WNSLVRIESLLGLSF-408 | 45.7 | 22 |
|  |  | 395-NSLVRIESLLGLSFS-409 | 15.4 | 23 |
|  |  | 397-LVRIESLLGLSFSKA-411 | 17.6 | 32 |
|  |  | 400-IESLLGLSFSKAAKR-414 | 13.8 | 25 |
|  |  | 401-ESLLGLSFSKAAKRR-415 | 19.0 | 24 |
|  |  | 462-ETPFHGIAPPRNKAV-476 | 13.8 | 22 |
|  |  | 515-VTGVRELRGKLVYRV-529 | 4.2 | 35 |
|  |  | 525-LVYRVKVDARTAAARC-539 | 21.8 | 26 |
|  |  | 563-GSLMRRVCPVNLTVG-577 | 36.1 | 30 |
|  |  | 571-PVNLTVGQTKRLQSI-585 | 31.7 | 24 |
|  |  | 576-VGQTKRLQSISADWV-590 | 8.4 | 23 |
|  |  | 579-TKRLQSISADWVVHV-593 | 28.7 | 30 |
|  |  | 587-ADWVVHVLTVSELDL-601 | 23.1 | 21 |
|  |  | 588-DWVVHVLTVSELDLE-602 | 18.2 | 24 |
|  |  | 590-VVHVLTVSELDLERT-604 | 39.8 | 22 |
| 1 | Dihydrolipoamide dehydrogenase | 3-RRNIAHLASYDVTVI-17 | 6.1 | 30 |
|  |  | 19-GGPGGYVAAIKAAQL-33 | 8.6 | 23 |
|  |  | 22-GGYVAAIKAAQLGLK-36 | 4.6 | 30 |
|  |  | 29-KAAQLGLKTACIEKR-43 | 26.5 | 22 |
|  |  | 50-CLNVGCIPSKALLHA-64 | 20.6 | 29 |
|  |  | 56-IPSKALLHATHLYHD-70 | 20.5 | 26 |
|  |  | 65-THLYHDAHANFAQYD-79 | 9.3 | 26 |
|  |  | 84-ENVTMDVSAMQAQKA-98 | 9.1 | 22 |
|  |  | 87-TMDVSAMQAQKAKGV-101 | 4.3 | 31 |
|  |  | 90-VSAMQAQKAKGVKAL-104 | 6.1 | 33 |
|  |  | 98-AKGVKALTGGVEYLF-112 | 27.3 | 25 |
|  |  | 114-KNKVTYYKGEGSFVN-128 | 23.5 | 27 |
|  |  | 116-KVTYYKGEGSFVNPN-130 | 15.3 | 29 |
|  |  | 165-FDEKVVMSSTGALDL-179 | 11.1 | 24 |
|  |  | 166-DEKVVMSSTGALDLD-180 | 26.1 | 24 |
|  |  | 168-KVVMSSTGALDLDHV-182 | 44.8 | 25 |
|  |  | 182-VPKKMIVVGGGVIGL-196 | 19.3 | 27 |
|  |  | 183-PKKMIVVGGGVIGLE-197 | 16.9 | 31 |
|  |  | 185-KMIVVGGGVIGLELG-198 | 34.8 | 20 |
|  |  | 190-GGGVIGLELGSVWAR-204 | 28.1 | 25 |
|  |  | 192-GVIGLELGSVWARLG-206 | 78.1 | 23 |
|  |  | 195-GLELGSVWARLGAEV-209 | 42.6 | 23 |
|  |  | 198-LGSVWARLGAEVTVV-212 | 6.0 | 24 |
|  |  | 199-GSVWARLGAEVTVVE-213 | 5.5 | 27 |
|  |  | 237-HEKIKIMTNTKVVSG-251 | 4.4 | 28 |
|  |  | 284-RPHTTGLNAEAINLQ-298 | 17.5 | 25 |
|  |  | 320-IGDVVNKGPMLAHKA-334 | 35.4 | 24 |
|  |  | 323-VVNKGPMLAHKAEEE-337 | 41.6 | 23 |
|  |  | 350-GHVNYNVIPGVIYTN-364 | 12.5 | 20 |
|  |  | 351-HVNYNVIPGVIYTNP-365 | 14.4 | 25 |
|  |  | 413-KKTDRILGVQIVCTA-427 | 42.3 | 22 |
| 2 | RNA helicase | 26-RRYLRTKRTTVARTR-40 | 12.4 | 25 |
|  |  | 39-TRRIREQPATALRTP-53 | 6.1 | 33 |
|  |  | 64-PALYPFFLPSHLCHL-78 | 42.7 | 25 |
|  |  | 76-CHLHCAIMSSDLADF-80 | 9.7 | 20 |
|  |  | 77-HLHCAIMSSDLADFD-81 | 9.8 | 23 |
|  |  | 93-DDVRTTVVAAQPMGV-107 | 13.4 | 23 |
|  |  | 94-DVRTTVVAAQPMGVG-108 | 9.8 | 23 |
|  |  | 96-RTTVVAAQPMGVGMG-110 | 6.0 | 25 |
|  |  | 103-AQPMGVGMGTHSAVAL-117 | 27.9 | 22 |
|  |  | 119-GFQDFCLKSELANAI-133 | 11.0 | 22 |
|  |  | 122-DFCLKSELANAIREN-136 | 10.4 | 25 |
|  |  | 147-QALPKAMLGADILAQ-161 | 14.2 | 23 |
|  |  | 148-ALPKAMLGADILAQA-162 | 15.5 | 26 |
|  |  | 169-TAVFVFALLEQVEKV-183 | 23.2 | 20 |
|  |  | 191-CQAVVLVHARELAYQ-205 | 31.9 | 33 |
|  |  | 213-FSKYLPYATTGVFFG-227 | 18.0 | 27 |
|  |  | 214-SKYLPYATTGVFFGG-228 | 35.8 | 22 |
|  |  | 235-VKQLKKEVPAIIVGT-248 | 34.4 | 25 |
|  |  | 242-VPAIIVGTPGRMKAL-256 | 11.7 | 22 |
|  |  | 250-PGRMKALIQNKAFDT-264 | 28.7 | 26 |
|  |  | 289-QEIFMKLPKEKQVMM-303 | 38.3 | 21 |
|  |  | 297-KEKQVMMFSATMTDE-311 | 14.0 | 22 |
|  |  | 324-TEIYVDQRAKLTLHG-338 | 5.1 | 26 |
|  |  | 333-KLTLHGLAQFYMNVT-347 | 17.9 | 24 |
|  |  | 412-ANNTRIMVATDLFGR-426 | 17.6 | 28 |
|  |  | 413-NNTRIMVATDLFGRG-427 | 27.0 | 21 |
| 11 | 2,4 dihydroxyhept-2-ene-1,7-dioic acid aldolase | 16-KPKFGIFLNSASPLL-30 | 4.2 | 21 |
|  |  | 17-PKFGIFLNSASPLLA-31 | 3.9 | 23 |
|  |  | 18-KFGIFLNSASPLLAG-32 | 3.7 | 25 |
|  |  | 19-FGIFLNSASPLLAGQ-33 | 3.9 | 24 |
|  |  | 20-GIFLNSASPLLAGQF-34 | 4.5 | 23 |
|  |  | 38-GYDWLLIDAQHSPVD-52 | 7.8 | 33 |
|  |  | 41-WLLIDAQHSPVDPLT-55 | 19.8 | 20 |
|  |  | 53-PLTMAHMIAAIRTGH-67 | 12.2 | 22 |
|  |  | 57-AHMIAAIRTGHSKVM-71 | 17.7 | 28 |
|  |  | 60-IAAIRTGHSKVMVRV-74 | 16.2 | 20 |
|  |  | 110-SCCYYPTTGTRSVYQ-124 | 8.7 | 27 |
|  |  | 111-CCYYPTTGTRSVYQP-125 | 11.6 | 26 |
|  |  | 123-YQPQQCMNAKGLLGY-137 | 31.6 | 25 |
|  |  | 134-LLGYVPESNKNVVVA-148 | 18.3 | 20 |
|  |  | 144-NVVVAFQVETAACIE-158 | 11.0 | 24 |
|  |  | 146-VVAFQVETAACIENL-160 | 5.4 | 26 |
|  |  | 157-IENLEEIMAVKGIDI-171 | 26.5 | 23 |
|  |  | 158-ENLEEIMAVKGIDIA-172 | 14.1 | 20 |
|  |  | 160-LEEIMAVKGIDIAFL-174 | 5.9 | 30 |
|  |  | 163-IMAVKGIDIAFLGQN-177 | 16.2 | 23 |
|  |  | 170-DIAFLGQNDLCMSMG-184 | 34.6 | 24 |
|  |  | 218-ILGLFLFGTDRVGEF-232 | 21.8 | 24 |
|  |  | 236-GFTFISIGSELHHAM-250 | 23.7 | 23 |
| 2 | Eukaryotic initiation factor 4a | 32-PLHQNLLRGIYSYGF-46 | 22.9 | 26 |
|  |  | 41-IYSYGFEKPSSIQQR-55 | 13.9 | 25 |
|  |  | 77-TGAFSIGLLQRLDFR-91 | 31.2 | 21 |
|  |  | 87-RLDFRHNLIQGLVLS-101 | 14.7 | 22 |
|  |  | 89-DFRHNLIQGLVLSPT-103 | 41.5 | 24 |
|  |  | 95-IQGLVLSPTRELALQ-109 | 20.2 | 25 |
|  |  | 102-PTRELALQTAEVISR-116 | 9.4 | 22 |
|  |  | 103-TRELALQTAEVISRI-117 | 7.9 | 31 |
|  |  | 138-QDDLRKLQAGVIVAV-152 | 5.2 | 36 |
|  |  | 139-DDLRKLQAGVIVAVG-153 | 6.4 | 24 |
|  |  | 146-AGVIVAVGTPGRVSD-160 | 22.6 | 24 |
|  |  | 147-GVIVAVGTPGRVSDV-161 | 47.5 | 22 |
|  |  | 161-VIKRGALRTESLRVL-175 | 34.5 | 22 |
|  |  | 192-IYEIFRFLPKDIQVA-206 | 19.4 | 23 |
|  |  | 193-YEIFRFLPKDIQVAL-207 | 23.1 | 21 |
|  |  | 205-VALFSATMPEEVLEL-219 | 15.4 | 24 |
|  |  | 259-MDLYETVSIAQSVIF-273 | 33.2 | 24 |
|  |  | 307-ERVMNTFRSGSSRVL-321 | 7.6 | 23 |
|  |  | 310-MNTFRSGSSRVLVTT-324 | 4.1 | 27 |
|  |  | 348-KENYLHRIGRGGRYG-362 | 22.3 | 21 |
|  |  | 376-VELLHEIEAHYHTQI-390 | 48.5 | 23 |
| 17 | Enolase | 19-TVEVELMTEAGVFRS-33 | 22.3 | 25 |
|  |  | 28-AGVFRSAVPSGASTG-42 | 6.1 | 28 |
|  |  | 29-GVFRSAVPSGASTGV-43 | 7.8 | 20 |
|  |  | 66-VKNVNEILAPALVGK-80 | 9.9 | 31 |
|  |  | 67-KNVNEILAPALVGKD-81 | 10.7 | 25 |
|  |  | 98-TKNKSKLGANAILGC-112 | 25.8 | 27 |
|  |  | 111-GCSMAISKAAAAKAG-125 | 12.9 | 27 |
|  |  | 113-SMAISKAAAAKAGVP-127 | 12.0 | 27 |
|  |  | 125-GVPLYRYIAGLAGTK-139 | 4.5 | 24 |
|  |  | 126-VPLYRYIAGLAGTKD-140 | 4.5 | 26 |
|  |  | 129-YRYIAGLAGTKDIRL-143 | 7.7 | 26 |
|  |  | 162-PFQEFMIAPTKATSF-176 | 5.8 | 23 |
|  |  | 163-FQEFMIAPTKATSFR-177 | 4.6 | 20 |
|  |  | 165-EFMIAPTKATSFREA-179 | 7.6 | 25 |
|  |  | 278-YERWVAEYPLVSIED-292 | 32.0 | 25 |
|  |  | 296-EDNFDEFSAITMALA-310 | 22.7 | 26 |
|  |  | 299-FDEFSAITMALAGKA-313 | 9.0 | 24 |
|  |  | 302-FSAITMALAGKAQIV-316 | 5.1 | 27 |
|  |  | 339-SLLLKINQIGTISES-353 | 39.7 | 20 |
|  |  | 362-ENGWSVMVSHRSGET-376 | 9.4 | 30 |
|  |  | 377-EDTYIADLSVGLGTG-391 | 18.9 | 26 |
| 8 | Protein transport protein Sec13 | 31-QFDYYGLQLATASSD-45 | 4.1 | 26 |
|  |  | 32-FDYYGLQLATASSDR-46 | 4.7 | 34 |
|  |  | 33-DYYGLQLATASSDRT-47 | 6.2 | 22 |
|  |  | 47-TIGIHVARAGAPLNR-61 | 23.9 | 24 |
|  |  | 59-LNRVATLTGHEGPVW-73 | 35.7 | 24 |
|  |  | 69-EGPVWMVSWAHPRFG-83 | 23.3 | 23 |
|  |  | 79-HPRFGNLLASASYDQ-93 | 4.5 | 34 |
|  |  | 128-PEEYGPVVATASSDG-142 | 33.0 | 33 |
|  |  | 164-SNQIAHAMGATSVTF-178 | 22.5 | 24 |
|  |  | 165-NQIAHAMGATSVTFA-179 | 7.6 | 24 |
|  |  | 175-SVTFAPFKSELVDHV-189 | 5.8 | 24 |
|  |  | 239-ASRFVILASCGQDKT-253 | 7.4 | 30 |
|  |  | 258-RKPWDQLCAEISEGA-272 | 8.9 | 34 |
|  |  | 278-WERSVIEFAEPVWRL-292 | 16.2 | 20 |
|  |  | 286-AEPVWRLSWAPSGEM-300 | 22.6 | 24 |
|  |  | 287-EPVWRLSWAPSGEML-301 | 14.3 | 21 |
|  |  | 289-VWRLSWAPSGEMLVV-303 | 20.9 | 22 |
|  |  | 297-SGEMLVVTNAKSEVF-311 | 25.4 | 23 |
| 8 | Cyclophilin 40 | 60-GCTFHRVIPGFMIQG-74 | 6.6 | 30 |
|  |  | 61-CTFHRVIPGFMIQGG-75 | 8.3 | 24 |
|  |  | 66-VIPGFMIQGGDFTNH-80 | 32.6 | 23 |
|  |  | 67-IPGFMIQGGDFTNHN-81 | 19.8 | 28 |
|  |  | 104-KSGLLAMANAGANTN-118 | 4.1 | 24 |
|  |  | 118-NGSQFFITTAPATHL-132 | 4.3 | 21 |
|  |  | 119-GSQFFITTAPATHLT-133 | 3.9 | 21 |
|  |  | 121- QFFITTAPATHLTGR-135 | 4.7 | 26 |
|  |  | 126-TAPATHLTGRHVVFG-140 | 23.8 | 26 |
|  |  | 132-LTGRHVVFGRVVRGM-146 | 20.8 | 23 |
|  |  | 140-GRVVRGMNTVRAVEQ-154 | 7.6 | 30 |
|  |  | 143-VRGMNTVRAVEQTPT-157 | 32.4 | 23 |
|  |  | 210-GESIRQIGNSHFKNA-224 | 26.6 | 25 |
|  |  | 259-CYNNHAMCAIKLQQW-273 | 8.1 | 23 |
|  |  | 291-AKAFFRRGTAALKAG-305 | 4.8 | 25 |
|  |  | 292-KAFFRRGTAALKAGD-306 | 4.9 | 20 |
|  |  | 332-SEAKEKVKAQKAKLA-346 | 7.8 | 25 |
|  |  | 335-KEKVKAQKAKLAANL-349 | 3.9 | 32 |
| 4 | Proteasome beta 2 subunit | 60-CRKIHYMAPNIMCCG-74 | 5.1 | 28 |
|  |  | 81-TEAVTNMVSSHLALH-95 | 6.7 | 29 |
|  |  | 82-EAVTNMVSSHLALHR-96 | 7.7 | 22 |
|  |  | 100-GKQSRVLEALTLLKR-114 | 10.9 | 27 |
|  |  | 113-KRHLYRYQGHVSAAL-127 | 6.3 | 24 |
|  |  | 114-RHLYRYQGHVSAALV-128 | 5.7 | 27 |
|  |  | 116-LYRYQGHVSAALVLG-130 | 7.6 | 24 |
|  |  | 117-YRYQGHVSAALVLGG-131 | 10.5 | 22 |
|  |  | 133-DVEGPFLATIAPHGS-147 | 7.7 | 23 |
|  |  | 136-GPFLATIAPHGSTDR-150 | 7.7 | 30 |
|  |  | 150-RLPFVTMGSGSIAAM-164 | 4.6 | 32 |
|  |  | 151-LPFVTMGSGSIAAMA-165 | 5.2 | 26 |
|  |  | 158-SGSIAAMAQLEVAYK-172 | 16.9 | 22 |
|  |  | 228-RQEVMLPPGTTPVLK-242 | 40.3 | 26 |
| 6 | Cyclophilin 2 | 13-ALSFLNVAAEPEVTA-27 | 10.9 | 24 |
|  |  | 28-KVYFDVMIDSEPLGR-42 | 31.9 | 24 |
|  |  | 29-VYFDVMIDSEPLGRI-43 | 13.7 | 22 |
|  |  | 41-GRITIGLFGKDAPLT-55 | 26.7 | 25 |
|  |  | 73-DSIFHRVIPNFMIQG-87 | 5.4 | 30 |
|  |  | 79-VIPNFMIQGGDFTNF-93 | 24.7 | 23 |
|  |  | 80-IPNFMIQGGDFTNFD-94 | 26.0 | 28 |
|  |  | 110-NLKVKHFVGALSMAN-124 | 11.0 | 25 |
|  |  | 111-LKVKHFVGALSMANA-125 | 8.8 | 24 |
|  |  | 114-KHFVGALSMANAGPN-128 | 17.0 | 24 |
|  |  | 131-GSQFFITTAPTPWLD-145 | 9.2 | 29 |
|  |  | 133-QFFITTAPTPWLDGR-147 | 19.8 | 23 |
|  |  | 149-VVFGKVLDGMDVVLR-163 | 45.0 | 25 |
|  |  | 170-NSHDRPVKPVKIVAS-184 | 46.3 | 23 |
| 10 | Prostaglandin f2-alpha synthase | 6-KAMVTLSNGVQMPQL-20 | 50.0 | 26 |
|  |  | 42-CAGYRHIDTAAIYKN-56 | 5.4 | 32 |
|  |  | 51-AAIYKNEESVGAGLR-65 | 42.3 | 28 |
|  |  | 135-EQLYKDKKVRAIGVS-149 | 30.3 | 20 |
|  |  | 137-LYKDKKVRAIGVSNF-151 | 16.9 | 27 |
|  |  | 148-VSNFHIHHLEDVLAM-162 | 23.2 | 21 |
|  |  | 150-NFHIHHLEDVLAMCT-164 | 10.0 | 25 |
|  |  | 199-PLGQGKLLSNPILAA-213 | 11.9 | 24 |
|  |  | 205-LLSNPILAAIGAKYN-219 | 24.5 | 29 |
|  |  | 208-NPILAAIGAKYNKTA-222 | 15.8 | 24 |
|  |  | 215-GAKYNKTAAQVILRW-229 | 4.9 | 29 |
|  |  | 226-ILRWNIQKNLITIPK-240 | 7.4 | 20 |
|  |  | 260-AEDVMRIDALNTNSR-274 | 18.9 | 22 |
|  |  | 263-VMRIDALNTNSRYGP-277 | 42.5 | 22 |
| 7 | Aldose-1-epimerase | 12-YDKLVWLETDVLKVG-26 | 37.0 | 32 |
|  |  | 20-TDVLKVGLTNYAASV-34 | 16.8 | 23 |
|  |  | 24-KVGLTNYAASVASIIQ-38 | 20.4 | 25 |
|  |  | 28-TNYAASVASIQVYHP-42 | 26.0 | 20 |
|  |  | 80-AGGVFTLDGVKYYTQ-94 | 16.2 | 25 |
|  |  | 113-HWGMKLIETANVIGV-127 | 21.1 | 31 |
|  |  | 149-TFIIDRSNPNALKTI-163 | 42.6 | 22 |
|  |  | 209-RNHWLRVPASRVAEA-223 | 4.0 | 32 |
|  |  | 210-NHWLRVPASRVAEAD-224 | 4.3 | 25 |
|  |  | 230-TGEFLSVEGTGLDFR-244 | 4.5 | 36 |
|  |  | 321-GQRYARWTGLLVGPQ-335 | 10.8 | 26 |
|  |  | 340-VANYYPKYPSCIVRR-354 | 12.6 | 24 |
|  |  | 341-ANYYPKYPSCIVRRG-355 | 21.1 | 26 |
| 14 | Hypothetical protein LinJ.19.1440 | 109-VIRDHFLKAVELNPN-123 | 9.7 | 25 |
|  |  | 111-RDHFLKAVELNPNDA-125 | 31.7 | 20 |
|  |  | 125-ATSLHCMGNWCFKIL-139 | 37.6 | 23 |
|  |  | 133-NWCFKILQIGWLERK-147 | 20.6 | 30 |
|  |  | 138-ILQIGWLERKAAALI-152 | 15.1 | 26 |
|  |  | 140-QIGWLERKAAALILG-154 | 8.8 | 26 |
|  |  | 141-IGWLERKAAALILGE-155 | 11.3 | 24 |
|  |  | 142-GWLERKAAALILGEP-156 | 17.4 | 20 |
|  |  | 147-KAAALILGEPPSSTY-161 | 23.8 | 20 |
|  |  | 148-AAALILGEPPSSTYE-162 | 19.5 | 24 |
|  |  | 163-ECLGYLLRSAEAGNT-177 | 8.8 | 23 |
|  |  | 196-EARKWYQKAIDMPAY-210 | 27.2 | 22 |
|  |  | 198-RKWYQKAIDMPAYTE-212 | 14.1 | 20 |
| 2 | RNA-binding protein | 20-TDNFRRNQTILFVG-34 | 4.6 | 29 |
|  |  | 21-DNFRRNQTILFVGN-35 | 5.5 | 23 |
|  |  | 25-RNQTILFVGNLPFQ-39 | 45.0 | 20 |
|  |  | 27-QTILFVGNLPFQTP-41 | 29.0 | 24 |
|  |  | 30-ILFVGNLPFQTPWQH-44 | 37.8 | 24 |
|  |  | 54-KVRYTDLIADRTGRP-68 | 10.6 | 27 |
|  |  | 70-GSALVTMMTVEGAEN-84 | 35.2 | 21 |
|  |  | 71-SALVTMMTVEGAENA-85 | 25.6 | 26 |
|  |  | 133-TAGGYYQGAAASGAA-147 | 7.2 | 23 |
|  |  | 135-GGYYQGAAASGAAAG-149 | 5.3 | 28 |
|  |  | 157-GSAYGRGAPYQVGAA-171 | 18.4 | 24 |
|  |  | 162-RGAPYQVGAAESTEM-176 | 30.3 | 23 |
| 9 | Activated protein kinase c receptor | 23-AGSYIKVVSTSRDGT-37 | 48.1 | 25 |
|  |  | 47-RHSVDSDYGLPSHRL-61 | 25.3 | 24 |
|  |  | 50-VDSDYGLPSHRLEGH-64 | 11.2 | 22 |
|  |  | 100-QRKFLKHTKDVLAVA-114 | 23.1 | 20 |
|  |  | 217-AALLWDLSTGEQLFK-231 | 31.5 | 22 |
|  |  | 218-ALLWDLSTGEQLFKI-232 | 12.9 | 27 |
|  |  | 227-EQLFKINVESPINQI-241 | 26.2 | 22 |
|  |  | 245-PNRFWMCVATERSLS-259 | 27.0 | 20 |
|  |  | 246-NRFWMCVATERSLSV-260 | 17.1 | 30 |
|  |  | 249-WMCVATERSLSVYDL-263 | 19.8 | 22 |
|  |  | 257-SLSVYDLESKAVIAE-271 | 12.0 | 32 |
| 13 | i/6 autoantigen-like protein | 21-EVLPRQMITVAALEA-35 | 35.3 | 22 |
|  |  | 24-PRQMITVAALEAGYC-37 | 43.6 | 30 |
|  |  | 27-MITVAALEAGYCLSS-41 | 40.6 | 25 |
|  |  | 33-LEAGYCLSSPTIGEA-47 | 13.9 | 22 |
|  |  | 80-AKCVVVVAPAHVITR-94 | 5.2 | 29 |
|  |  | 81-KCVVVVAPAHVITRR-95 | 5.4 | 25 |
|  |  | 94-RRSLEEIMAKGSSKK-108 | 44.0 | 33 |
|  |  | 134-KDFMRALYGDLGVRC-148 | 13.9 | 26 |
|  |  | 143-DLGVRCLAARRKLDA-158 | 18.7 | 27 |
|  |  | 149-LAARRKLDALEAKRR-163 | 13.0 | 28 |
|  |  | 152-RRKLDALEAKRREQE-166 | 11.1 | 26 |
| 3 | GTP-binding protein | 35-EFEKRYVATVGVDVH-49 | 9.7 | 24 |
|  |  | 37-EKRYVATVGVDVH-51 | 6.3 | 27 |
|  |  | 76-LRDGYYVEGQCAIIM-90 | 30.9 | 23 |
|  |  | 79-GYYVEGQCAIIMFDV-93 | 48.0 | 30 |
|  |  | 126-DCADRQVKAKMITFH-140 | 14.9 | 25 |
|  |  | 144-GLQYYDISAKSNYNF-158 | 5.7 | 25 |
|  |  | 159-EKPFVWLAKKLANDP-173 | 31.5 | 28 |
|  |  | 181-PMLDTDVVALTAEQV-195 | 24.0 | 23 |
|  |  | 184-DTDVVALTAEQVAAL-198 | 5.2 | 33 |
|  |  | 192-AEQVAALEAWQQAMA-206 | 26.1 | 25 |
| 5 | Ribonucleoprotein p18, mitochondrial precursor | 1-MRRLSSQLMCTAAAA-15 | 9.7 | 22 |
|  |  | 3-RLSSQLMCTAAAARF-17 | 11.4 | 21 |
|  |  | 6-SQLMCTAAAARFASA-20 | 9.4 | 24 |
|  |  | 12-AAAARFASAGGAKKY-26 | 21.5 | 21 |
|  |  | 14-AARFASAGGAKKYDL-28 | 20.7 | 20 |
|  |  | 27-DLFGYEVDTNTALWI-41 | 29.5 | 20 |
|  |  | 46-KCRYYDEAGEVLVNM-60 | 13.3 | 26 |
|  |  | 131-QFRLGYCVAKLMEAE-145 | 33.7 | 25 |
|  |  | 143-EAEFKRVPTELVQQN-157 | 5.4 | 31 |
| 12 | Phosphomannomutase | 27-KQTLAKVRAAGFKLG-41 | 10.2 | 34 |
|  |  | 35-AAGFKLGVVGGSDFA-49 | 41.9 | 22 |
|  |  | 60-LEDFDYVFSENGLLA-74 | 16.1 | 22 |
|  |  | 62-DFDYVFSENGLLAYK-76 | 7.2 | 21 |
|  |  | 63-FDYVFSENGLLAYKD-77 | 7.2 | 24 |
|  |  | 84-NSLLKALGNEKVVAF-98 | 5.2 | 27 |
|  |  | 100-KKCLHLIADLDIPVQ-114 | 37.5 | 23 |
| 15 | Pyrroline-5-carboxylate reductase | 49-LYQVRSVSAVELAEQ-63 | 7.0 | 22 |
|  |  | 63-QSDIIMLGVKPYGIV-77 | 11.3 | 21 |
|  |  | 130-VTSISGNSAVTLDDE-144 | 23.0 | 20 |
|  |  | 147-VVKLFSAIGKAYLVA-161 | 8.5 | 20 |
|  |  | 148-VKLFSAIGKAYLVAE-162 | 9.4 | 24 |

^a^ID – The numbers correspond to the specific spots indicated in Fig 2.

^b^MHC class II-restricted peptides were chosen based on their ability to bind with high affinity (NetMHCII: ≤50nM, SYFPEITHI: ≥20) to the HLA-DRB1 allele.
